# Freshwater invertebrate responses to fine sediment stress: A multi‐continent perspective

**DOI:** 10.1111/gcb.17084

**Published:** 2023-12-09

**Authors:** Morwenna McKenzie, Andrew Brooks, Marcos Callisto, Adrian L. Collins, Jessica M. Durkota, Russell G. Death, J. Iwan Jones, Marden S. Linares, Christoph D. Matthaei, Wendy A. Monk, John F. Murphy, Annika Wagenhoff, Martin Wilkes, Paul J. Wood, Kate L. Mathers

**Affiliations:** ^1^ Geography and Environment Loughborough University Loughborough UK; ^2^ Department of Planning and Environment, Surface Water Science NSW Government Wollongong New South Wales Australia; ^3^ Laboratory of Ecology of Benthos, Department of Genetics, Ecology and Evolution Institute of Biological Sciences, Federal University of Minas Gerais Belo Horizonte Brazil; ^4^ Net Zero and Resilient Farming, Rothamsted Research Okehampton Devon UK; ^5^ Environment Agency Worthing UK; ^6^ Innovative River Solutions, School of Agriculture and Environment Massey University Palmerston North New Zealand; ^7^ School of Biological and Behavioural Sciences Queen Mary University of London London UK; ^8^ Department of Zoology University of Otago Dunedin New Zealand; ^9^ Faculty of Forestry and Environmental Management Environment and Climate Change Canada, Canadian Rivers Institute, University of New Brunswick Fredericton New Brunswick Canada; ^10^ Cawthron Institute Nelson New Zealand; ^11^ School of Life Sciences University of Essex Colchester UK

**Keywords:** aquatic biodiversity, community composition, conservation, ecological threshold, ecosystem function, global scale

## Abstract

Excessive fine sediment (particles <2 mm) deposition in freshwater systems is a pervasive stressor worldwide. However, understanding of ecological response to excess fine sediment in river systems at the global scale is limited. Here, we aim to address whether there is a consistent response to increasing levels of deposited fine sediment by freshwater invertebrates across multiple geographic regions (Australia, Brazil, New Zealand and the UK). Results indicate ecological responses are not globally consistent and are instead dependent on both the region and the facet of invertebrate diversity considered, that is, taxonomic or functional trait structure. Invertebrate communities of Australia were most sensitive to deposited fine sediment, with the greatest rate of change in communities occurring when fine sediment cover was low (below 25% of the reach). Communities in the UK displayed a greater tolerance with most compositional change occurring between 30% and 60% cover. In both New Zealand and Brazil, which included the most heavily sedimented sampled streams, the communities were more tolerant or demonstrated ambiguous responses, likely due to historic environmental filtering of invertebrate communities. We conclude that ecological responses to fine sediment are not generalisable globally and are dependent on landscape filters with regional context and historic land management playing important roles.

## Graphical abstract

We investigated whether there is a consistent response to increasing levels of deposited fine sediment by freshwater invertebrates across multiple geographic regions (Australia, Brazil, New Zealand, and the UK). Results indicated that ecological responses are not globally consistent and are instead dependent on both the region and the facet of invertebrate diversity considered, that is, taxonomic or functional trait structure. We conclude that ecological responses to fine sediment are not generalisable globally and are dependent on landscape filters with regional context and historic land management playing important roles.

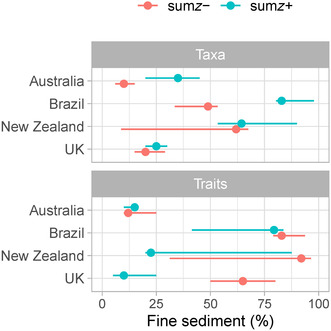

## 1 INTRODUCTION

Globally, freshwater systems are under significant pressure from anthropogenic stressors. Declines of biodiversity in aquatic environments are disproportionate, far exceeding those of terrestrial and marine systems (Higgins et al., 2021; Reid et al., 2019). In particular, excessive delivery and deposition of fine sediment (particles <2 mm) in river systems represents a major global threat to aquatic ecosystem health with inputs of fine sediment now far exceeding natural background levels (Dudgeon, 2019; Foster et al., 2011). Anthropogenic activities such as agricultural intensification, mining and deforestation have all increased the supply of fine sediments to rivers (Collins et al., 2009). Moreover, excessive transport and delivery of fine sediment is expected to be further exacerbated by changes to rainfall and run‐off regimes under future climatic change (Burt et al., 2016). Tackling the ecological implications of fine sediment is therefore a global and urgent issue, which continues to present ongoing challenges for monitoring and regulatory agencies.

The effects of excessive fine sediment deposition in rivers span multiple trophic levels (Owens et al., 2005). However, assessment of the effects of fine sediment, as with other anthropogenic stressors, is often performed using invertebrate communities as a proxy for wider ecosystem health and habitat quality. Fine sediment can affect invertebrate communities through a variety of mechanisms. The settling and infiltration of fine sediment in gravel beds reduces habitat heterogeneity, clogs interstitial pore space and limits intragravel flows and hydraulic connectivity (Dubuis & De Cesare, 2023; Wharton et al., 2017; Yarnell et al., 2006). This in turn alters the suitability of the substrate for some invertebrate taxa, impacts the exchange of oxygen and removal of excreta, increases invertebrate dispersal via drift and can affect respiration and feeding activities, which ultimately shapes the biodiversity and structure of invertebrate communities (Brown et al., 2019; Descloux et al., 2014; Mathers et al., 2017; Piggott et al., 2015).

Traditionally, taxonomic approaches have been used to quantify invertebrate responses to fine sediment pressures (Gieswein et al., 2019; Turley et al., 2015). However, quantifying community responses using functional traits can provide new insights into the mechanisms behind change occurring, rather than simply observing that a change has occurred (Culp et al., 2011). Consequently, we can infer ecosystem functioning (or lack of) from a combination of functional diversity metrics (Cadotte et al., 2011; Díaz et al., 2013). Individual traits that are sensitive are filtered by the prevailing environmental conditions (i.e., environmental filtering), which in turn shapes the functional assemblage of the invertebrate community (Floury et al., 2017). At the community level, some loss of functioning associated with fine sedimentation has been reported from a limited number of studies (Lange et al., 2014). However, until recently, trait‐based approaches were limited to the geographic region within which the trait databases were developed. Inconsistencies between trait groupings (e.g., feeding group) and individual trait modalities (e.g., shredder, filterer and scraper), as well as variation in taxonomic resolution, have meant multi‐country or region analyses has been limited (but see Brown et al., 2018; Mathers et al., 2022). With a new harmonised trait database (Kunz et al., 2022) available, it is possible for the first time to analyse functional responses to fine sediment at a multi‐continental spatial scale.

Despite the deleterious effects of excessive fine sediment deposition on river biodiversity, thresholds (such as standards or guidelines) are rarely recognised in management policies, unlike for example flow requirements (Directive 2000/60/EC of the European Parliament and of the Council Establishing a Framework for the Community Action in the Field of Water Policy, 2000). Where available, regulatory guidelines are not always based on explicit evidence of ecological degradation (Mondon et al., 2021) and are not necessarily thresholds at which populations or communities change abruptly or shift to alternate stables states (*sensu* the definition of ecological threshold by Groffman et al., 2006). Ecological threshold analysis, which investigates such abrupt changes, could therefore represent a valuable tool to aid in the establishment of management thresholds for fine sediment (Groffman et al., 2006). The concept of an ecological threshold is founded on species tolerating the stressor up to a threshold point in which the effects become nonlinear, or disproportionate, relative to further increases in the stressor (D'Amario et al., 2019; May, 1977). Threshold analysis has been successfully applied to a variety of different environmental drivers, or stressors (e.g., excess nutrients, urbanisation and river flow) and species groups (e.g., birds, fish, diatoms, algae, macrophytes and invertebrates; Chen & Olden, 2020; Sonderegger et al., 2009; Wagenhoff et al., 2017) but, as yet, fine sediment pressures have not been examined extensively. Defining ecological thresholds has recently drawn scientific debate, with concerns about the fundamental ability to identify thresholds from empirical data, and whether targets based on thresholds are detrimental to management practices (Hillebrand et al., 2020, 2021). However, the identification of safe operating spaces, or acceptable limits of change, represents a valuable tool for the management of environmental stressors (Lade et al., 2021), and provides a vector to interpret complex ecological responses across pressure gradients.

The limited existing research, which has sought to quantify invertebrate ecological thresholds to deposited fine sediment, presents varying and conflicting results (Burdon et al., 2013; Kaller & Hartman, 2004; Paul & McDonald, 2005; Schäffer et al., 2020). This can be attributed to inconsistencies in the ecological response variable (i.e., taxonomic level or community facet), measurement of fine sediment and statistical methods applied. These studies are typically based on small spatial scales meaning scaling up is not possible. As such, there remains a crucial need for studies to establish the thresholds at which ecological degradation occurs across the deposited sediment gradient in order to fully characterise the ecological implications. This study represents the first multicontinental perspective in identifying country‐specific deposited fine sediment thresholds. Through a combination of taxonomic and functional analyses, we sought to assess riverine invertebrate responses to fine sediment on a global scale. Specifically, we tested the following: (1) whether taxonomic and functional facets of invertebrate communities vary in response to deposited fine sediment across geographic regions and (2) if there are globally consistent associations of invertebrate community metrics (biological indicators) with deposited fine sediment.

## 2 METHODS

### 2.1 Data sets characterised

Data were obtained from three continents constituting the four countries of Australia, Brazil, the UK (collective) and New Zealand (Figure S1 in Data S1). Data comprised paired biological (invertebrates) and environmental (visual fine sediment cover %) data collected during the same sampling occasion (Table 1) consisting of a total of 6491 samples. Substrate composition was determined at the same spatial scale as biological sampling via visual estimates of percentage cover of the bed surface at sample reaches across a range of size classes (e.g., boulder, cobble and gravel), with the percentage of fine sediment calculated by aggregating all substrate categories <2 mm in diameter (clay, silt and sand). Visual estimates have been shown to be a reliable proxy for fully quantitative methods of assessing fine sediment in aquatic systems, with low inter‐operator variability (Conroy et al., 2016a; Mckenzie et al., 2022b). Fine sediment data were initially assessed to ensure a suitable gradient coverage prior to analysis (other data sets were excluded based on this assessment). Invertebrates were collected using standard quantitative methodologies (kick or Surber sampling) and identified to either family or mixed‐taxon level. Data were converted to relative abundance to ensure comparability (e.g., Chen & Olden, 2020) and resolved at family level to account for the mixed levels of identification (Everall et al., 2017; Stubbington et al., 2022). Sites were filtered (either prior to acquisition or during data collection) to ensure a reduction in co‐occurring stressors, which may confound or interact with the effects of fine sediment. Detailed descriptions of sampling methods and data filtering for each source can be found in Data S1.

**TABLE 1:** Summary of data sources in study.

Country and source publications/databases	Duration of record	Number of samples^a^	Number of taxa
Australia (New South Wales) (Compiled by the New South Wales Department of Planning and Environment, 2018)	1994–2011	3099	174
Brazil (Cerrado Biome) (Agra et al., 2021; Callisto et al., 2019; Castro et al., 2017, 2018, 2020; Firmiano, Canedo‐Argüelles, et al., 2021; Firmiano, Castro, et al., 2021; Macedo et al., 2018; Silva et al., 2018, 2021)	2009–2017	232	102
UK (Environment Agency, 2021; Jones et al., 2017; Murphy et al., 2015, 2017)	1990–2019	2818	173
New Zealand (Hunter, 2020; Lange et al., 2014; Magbanua et al., 2010; Matthaei et al., 2006; Wagenhoff et al., 2011)	2003–2011	342	108

^a^ After data filtering to reduce confounding factors.

Functional trait databases providing harmonised trait data for Europe, North America (used for Brazil), New Zealand and Australia at the family level were compiled (Kunz et al., 2022). A total of 22 individual trait modes across six trait categories were used in the analyses (Table S3 in Data S1). The number of taxa assigned traits varied by country: Australia (78), Brazil (44), the UK (79) and New Zealand (59). The trait database was acquired as proportional traits (rather than fuzzy coded traits).

### 2.2 Statistical analysis

Gradient Forest (GF) analysis (*gradientForest* package) (Ellis et al., 2012) was applied to compare compositional change (or turnover) across the deposited sediment gradient to define community threshold points. GF is an extension of random forest (Breiman, 2001) and applies a regression tree approach to quantify thresholds using nonlinear responses across an environmental gradient. First, separate random forests (ntree = 1000) are constructed for each species (or trait modality for functional community analysis). Next, GF aggregates the split value of each individual tree and their fit improvement across all species with positive fits (defined as *R* ^2^ > 0). When quantifying the overall compositional change across the gradient, each split in the GF contributes relative to its fit improvement, and each species contributes relative to its variance (as *R* ^2^) explained by the environmental predictor(s) (Chen & Olden, 2020; Compton et al., 2013; Ellis et al., 2012). One of the benefits of GF is that it is unaffected by unevenly distributed data across the environmental gradient, which is often the case with field survey data, as it standardises split density by the density of observed values across the gradient (Ellis et al., 2012; Wagenhoff et al., 2017). With the standardisation expressed as a ratio, points where the value is >1 represent areas where compositional change is highest compared with the turnover occurring elsewhere across the gradient, thus indicating community threshold points. All threshold points were identified using the R package *pracma* (Borchers, 2019; Chen & Olden, 2020).

Threshold Indicator Taxa ANalyses (TITAN) (Baker & King, 2013) were carried out using the *TITAN2* package (Baker et al., 2015). TITAN is a nonparametric method that uses a resampling technique to detect abrupt change points of abundance and occurrence across an environmental gradient (Baker & King, 2013; King & Baker, 2010). TITAN determines these change points for individual components of the community which are either sensitive (respond negatively) or tolerant (respond positively) to an environmental gradient, providing further information on the community thresholds than identified by GF alone. A taxon or trait is identified as either responding positively (*z*+) or negatively (*z*−) to the deposited sediment gradient if; (a) the change in frequency and abundance is the same for ≥95% of all bootstrap samples (i.e., pure) and, (b) ≥95% of all bootstrap samples are significantly different from a random distribution (*p* < .05) (i.e., reliable). The sum of all IndVal *z* scores (sum*z*) can be used as an indicator of taxonomic or functional community level threshold by identifying peaks along the gradient associated with the maximum decline or increase in frequency and/or abundance of negative and positive responders, respectively (King et al., 2016; Monk et al., 2018). Function parameters were set as 250 random permutations (*numPerm*) and 500 bootstrap replicates (*nBoot*) (Khamis et al., 2014; Lencioni, 2018; Porter‐Goff et al., 2013).

**TABLE 2:** Percentage of individual taxa or trait modalities identified as indicators of deposited fine sediment across analysis methods: TITAN (pure and reliable taxa only where ≥95% of 999 bootstrap runs are significantly different from a random distribution where *p* < .05) and Gradient Forest (*R* ^2^ > 0).

Analysis	Community	Australia	Brazil	New Zealand	UK
TITAN	Taxa *z*−	30%	30%	19%	17%
	*z*+	39%	5%	30%	52%
	Traits *z*−	62%	33%	40%	29%
	*z*+	38%	33%	20%	52%
Gradient Forest	Taxa	32%	2%	21%	18%
	Traits	82%	5%	45%	45%

Ten taxonomic and functional metrics were calculated to determine the consistency of responses in commonly employed biodiversity metrics to fine sediment. Taxonomic indices calculated were taxa richness, Ephemeroptera, Plecoptera and Trichoptera (EPT) richness, EPT index (EPT richness as a proportion of taxa richness), Simpson's diversity index, and Pielou's evenness. Five functional diversity indices were calculated using the *FD* package (Laliberté et al., 2014) comprising functional richness (FRic), functional dispersion (FDis), functional evenness (FEve), functional divergence (FDiv) and Rao's quadratic entropy (RaoQ). Spearman's rank correlation (due to non‐normal data distribution) assessed the performance of the indices against visual fine sediment (%). Pairwise correlations were corrected for multiple comparisons using the Holm–Bonferroni correction (Holm, 1979). All analyses were conducted in the R environment (R Development Core Team, 2022).

## 3 RESULTS

### 3.1 Community responses to deposited fine sediment

**FIGURE 1:**
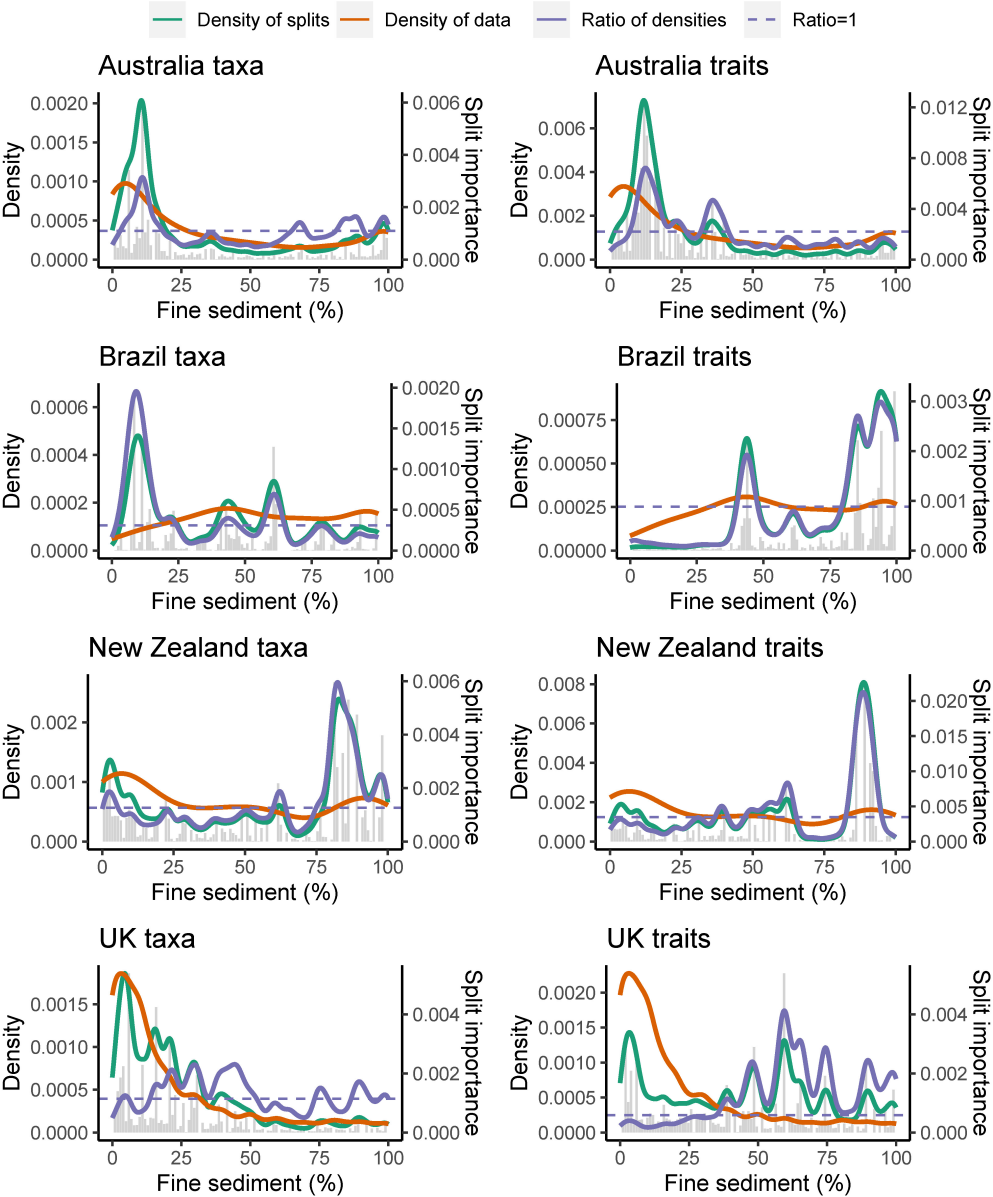
Taxonomic (taxa) and functional (traits) change in invertebrate communities across a deposited fine sediment gradient (% visual cover) as identified by Gradient Forest analysis. Split density importance (grey bars) shown on secondary y axis (right hand side). Points along the gradient where the ratio of densities >1 indicate areas where compositional change is highest compared with the turnover occurring elsewhere across the gradient, thus indicating community threshold points.

Thresholds in taxonomic responses were identified by GF to occur at low fine sediment coverage for Australia and Brazil, occurring at <12% cover (see peaks in the ratio densities [purple line] in Figure 1; Table S1 in Data S2). The UK taxonomic responses exhibited multiple thresholds across a broad range of fine sediment coverage (16%–99%) with the peak threshold occurring at 44% cover. New Zealand exhibited the lowest threshold for all countries (3% cover) but had a peak taxonomic threshold at 89% coverage. Thresholds in functional community responses for Australia and New Zealand closely mirrored their respective taxonomic peak threshold points at 12% and 89%, respectively (Figure 1; Table S1 in Data S2). In Brazil, a high functional threshold was observed at 94% in contrast to the low taxonomic threshold at 9%. In the UK, multiple functional thresholds were observed.

**FIGURE 2:**
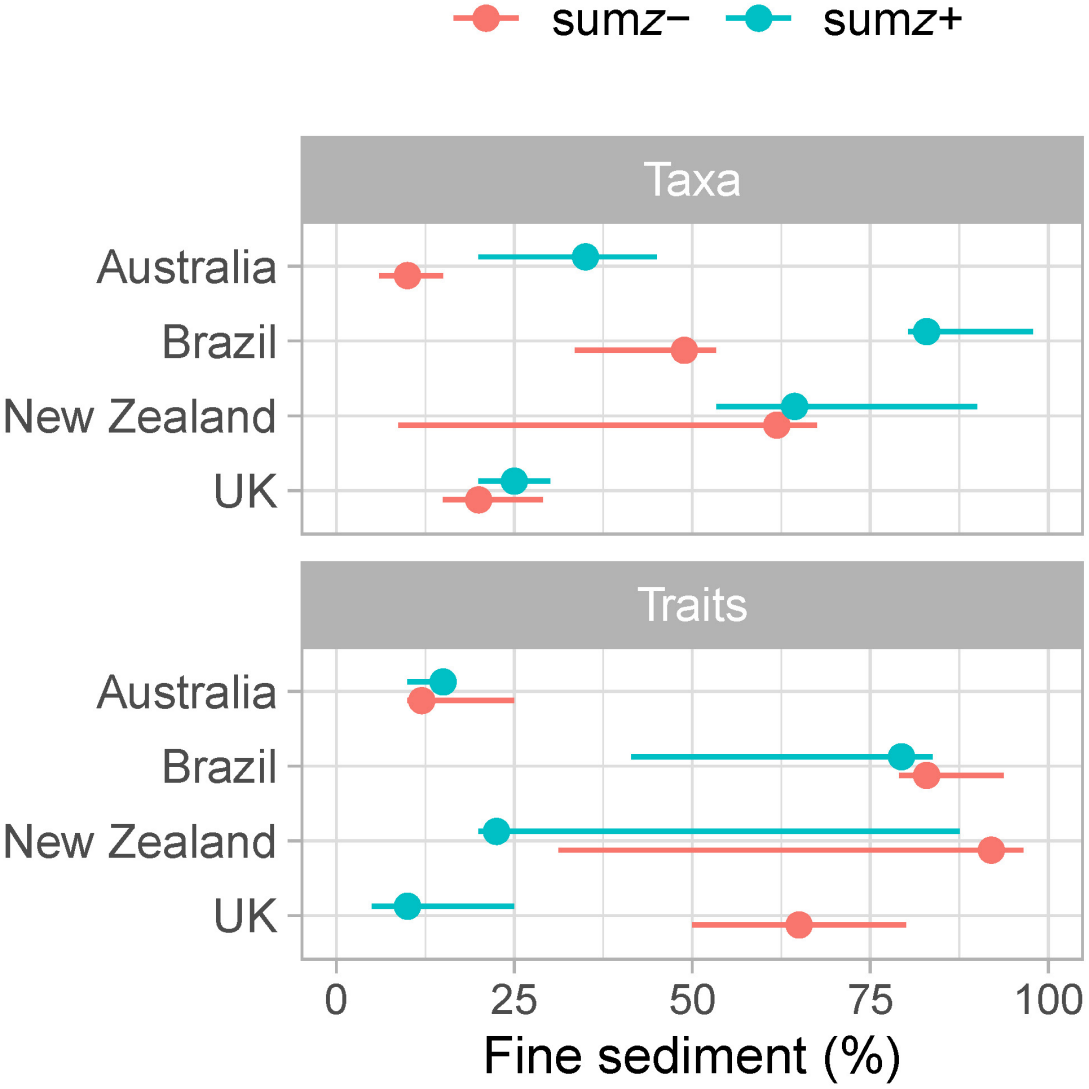
Observed sum*z*− (red) and sum*z*+ (blue) maxima (i.e., change points) identified by Threshold Indicator Taxa ANalysis (TITAN) for taxonomic (taxa) and functional (traits) measures of communities. Peak change points indicated as circles, with 5th and 95th percentile distributions as horizontal lines. Change points are filtered to include only pure and reliable taxa/traits.

**FIGURE 3:**
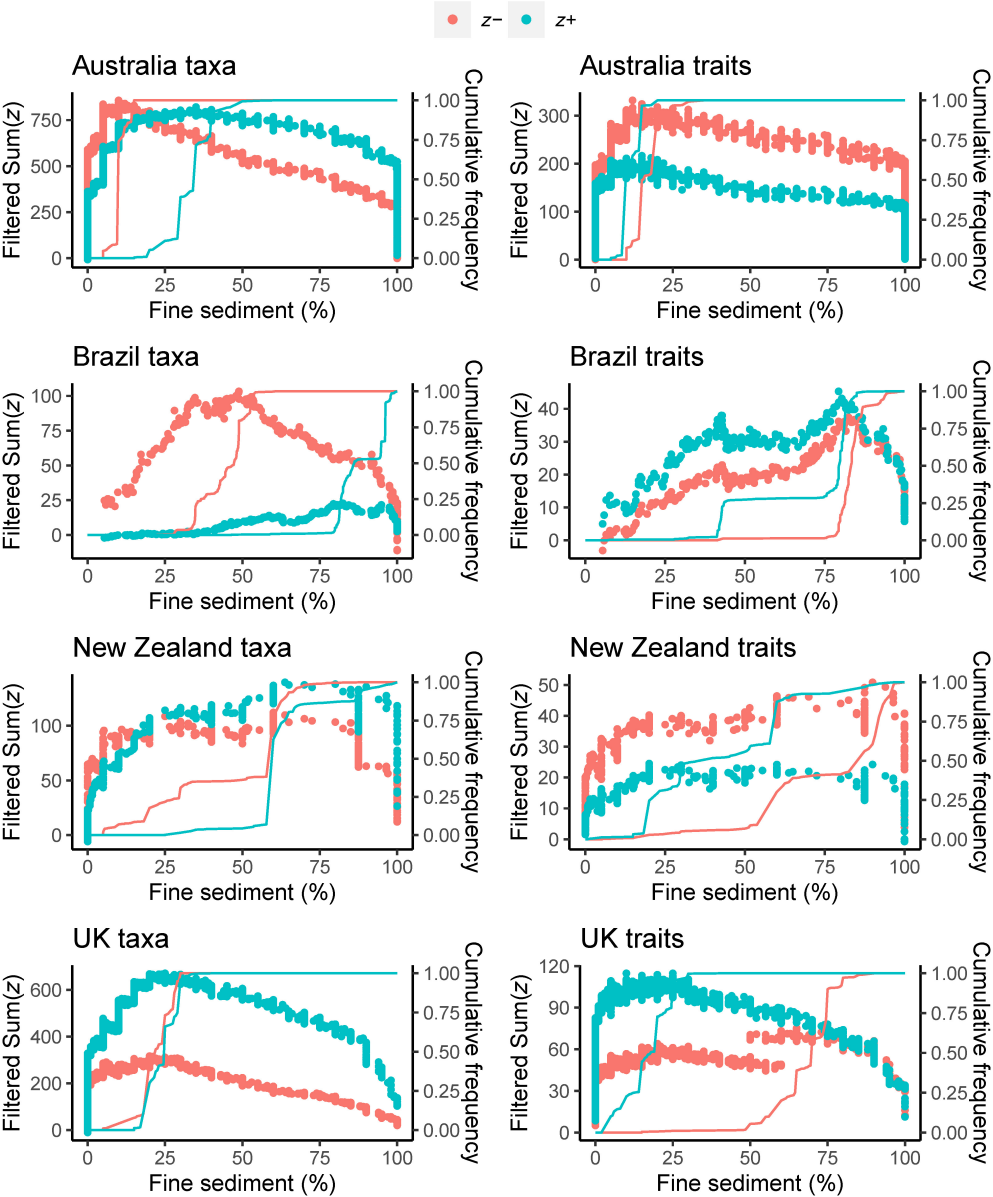
Taxonomic (taxa) and functional (traits) community change identified by Threshold Indicator Taxa ANalysis (TITAN) shown as density plots of sum*z* for positively responding taxa (blue circles) and negatively responding taxa (red circles) across fine sediment (%) gradient for Australia, Brazil, New Zealand and the UK. Peaks in values across the sum*z* gradient indicate points of large amounts of community change. The gradient of the cumulative frequency distribution of sum*z*− and sum*z+* indicates the certainty of change point locations with vertical lines indicating higher confidence relative to shallow gradient lines. Sum*z* are filtered to include only pure and reliable taxa.

When TITAN analyses were considered, taxonomic and functional measures showed contrasting change points along the deposited fine sediment gradient (Figures 2 and 3). Sensitive (i.e., negatively responding, *z‐*) functional traits were associated with higher fine sediment thresholds than sensitive taxa for all countries (Figure 2). Sensitive taxa generally had a lower threshold point than tolerant (i.e., positively responding, *z*+) taxa. By contrast, tolerant traits had a lower threshold point than sensitive traits (except in Australia). However, observing peaks in the sum*z* scores across the deposited sediment gradient provides more information than observing a single change point location alone (Figure 3). Peaks in sum*z* density at <10% deposited sediment were present for sensitive taxa in Australia, which was the single lowest taxonomic changepoint identified across all countries. Sensitive taxa in Brazil displayed a single peak, which occurred between 30% and 60% fine sediment coverage. Both sensitive and tolerant traits in Brazil peaked at the upper end of the gradient, with the rate of change increasing steadily along the gradient. In contrast, both sensitive and tolerant taxa in the UK displayed peaks at the lower end of the deposited sediment gradient (~20%–25%). Tolerant traits in the UK demonstrated peaks towards the lower end of the gradient (<20%), whilst in contrast, sensitive traits peaked at the upper end (75%). There were no distinctive peaks for New Zealand for either taxonomic or functional responses. When assessing overall community change, the narrower quartile ranges (Figure 2), and near‐vertical cumulative frequency distribution plots (Figure 3) indicated that there was a higher degree of confidence in the changepoint locations for both taxonomic and functional measures in Australia and the UK relative to Brazil and New Zealand.

### 3.2 Ecological indicators of deposited fine sediment

**FIGURE 4:**
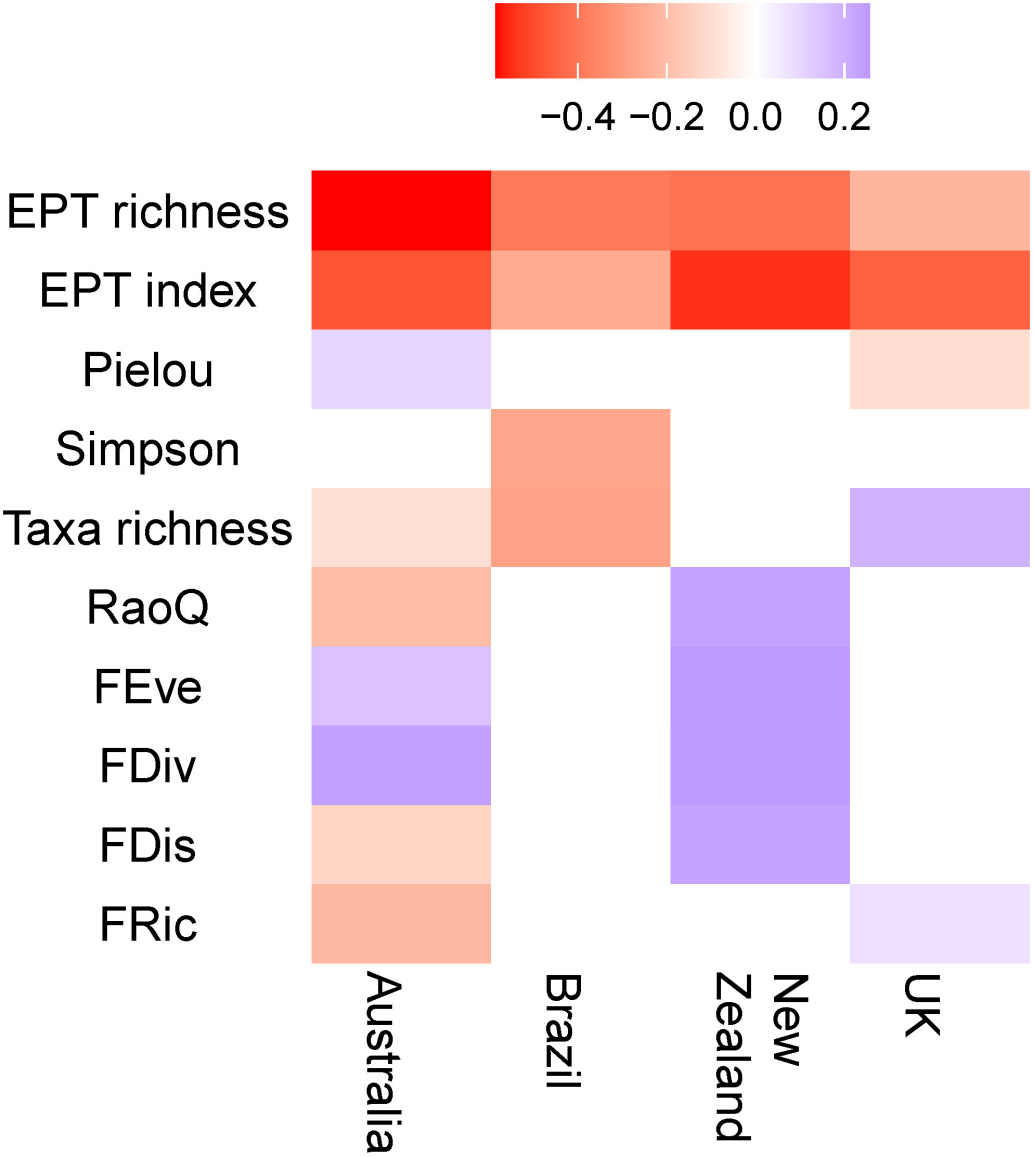
Correlation matrix for taxonomic and functional indices of community composition with deposited fine sediment (% visual cover) for each country. Colour ramp indicates Spearman's rank correlation coefficient. Only significant pairwise correlations (*p* < .05) are presented (Holm–Bonferroni corrected).

Ephemeroptera, Plecoptera and Trichoptera indices (EPT richness and EPT index) were found to be significantly negatively correlated with deposited fine sediment coverage in all four countries (Figure 4; full statistical outputs available in Table S2 in Data S2). The correlations were strongest for Australia, with EPT richness and fine sediment coverage being the strongest pairwise correlation across all combinations. Overall, at the family‐level taxonomic resolution, the UK had the highest EPT richness compared with all other regions, whilst in New Zealand, both the lowest number of taxa (across all orders) and EPT richness (Figure S1 in Data S2) were recorded. In Brazil, the lowest EPT index scores were found despite taxonomic richness being the highest, indicating the sites are taxonomically rich but with a low proportion of EPT taxa. Taxonomic richness was significantly correlated with deposited fine sediment coverage in three countries, in a negative fashion in Australia and Brazil, but positively in the UK. Simpson's diversity index was overall poorly correlated with deposited fine sediment, with a significant negative correlation for only one country (Brazil). Multiple functional indices were significantly correlated with fine sediment in Australia and New Zealand, but only FRic demonstrated a significant correlation for the UK and no significant correlations were observed in Brazil (Figure 4).

Overall, our findings demonstrate that based on indicator taxa as identified by TITAN (i.e., either *z*− or *z*+), taxonomic change in Brazil appeared to be driven by negatively responding taxa, whereas the UK was driven by positively responding taxa (Table 1). New Zealand and Australia demonstrated a more even spilt between negatively and positively responding taxa. Functional change identified by TITAN indicator traits found a greater proportion of positively responding traits in the UK, with Australia and New Zealand compositional change being driven by negatively responding traits (Table 2). Brazil demonstrated the same proportion (33%) of negatively and positively responding traits (Table 2). Considering GF analysis, a low proportion of taxa or traits were identified in Brazil indicating that the compositional change of only a few taxa (2%)/traits (5%) could be predicted by the deposited fine sediment gradient, whilst Australia demonstrated a high proportion of traits (82%) associated with compositional change over the fine sediment gradient (Table 2;Tables S3–S11 in Data S2 for full results).

## 4 DISCUSSION

Excess fine sediment deposition is a pervasive stressor in aquatic environments causing complex, often nonlinear, impacts on freshwater communities. Most existing research quantifying ecological threshold responses to deposited fine sediment is based on small spatial scales (e.g., Larsen et al., 2009). We have, for the first time, quantified at what point along the gradient of % deposited fine sediment cover, disproportionately large impacts occur for invertebrate communities across multiple continents. Compared with other gradients (e.g., urbanisation and glacier loss) in which consistent responses are evident across multiple geographic regions (Brown et al., 2018; Chen & Olden, 2020), the results of our study suggest that biotic responses to deposited fine sediment are dependent on both the region and the facet of the invertebrate community (e.g., taxonomic or functional) considered.

The most consistent responses to fine sediment were evident for Australia and the UK. For Australia, fine sediment appeared to cause the most severe taxonomic changes when the sediment coverage was low (i.e., <25%). In contrast to the UK, widespread conversion of forested land to agriculture has only occurred relatively recently in Australia, since European colonisation in the late 18th century, with the greatest rates of forest loss and degradation occurring since the 1970s (Allen, 1999; Bradshaw, 2012). This more recent land use change may have resulted in a greater number of extant sensitive species in Australia relative to the UK, thus driving a strong community response at low deposited sediment % cover. Despite a similar timescale of land conversion occurring in New Zealand as in Australia, agricultural land cover in the sampled catchments was greater than in Australia (Table S2 in Data S1), likely resulting in invertebrate communities composed of taxa more tolerant to fine sediment from continuous exposure (similar to the UK and Brazil). In New Zealand, a large proportion of sampled sites were at the high end of the deposited fine sediment coverage range (Figure S1 in Data S1), and overall lower numbers of pollution‐sensitive EPT taxa were found in this region compared to other countries (Figure S2 in Data S2). As such, it may explain why, for New Zealand, we found the greatest rate of community change for both the taxonomic and functional responses to be at the higher end of the deposited sediment gradient. This suggests that for the stream types sampled, environmental filtering has already taken place. In other words, the taxa/trait communities have been filtered by the prevailing environmental conditions which we speculate could be associated with pervasive and longstanding fine sediment inputs. However, the wide confidence intervals for the change points identified by TITAN in New Zealand suggest uncertainty of a specific community threshold point and an ambiguous ecological response to fine sediment which may reflect context specificity of fine sediment stress.

Taxonomic and functional responses in Brazil were either poorly related or unaffected by fine sediment. Although the threshold points identified by GF were at low deposited fine sediment coverage, only two taxa demonstrated positive *R* ^2^ values in the model. Therefore, these threshold points represent the turnover of two of 102 taxa in total (Table 1; Tables S3 and S4 in Data S2). Whilst this finding could be attributed to the lower sample size (with Brazil representing the smallest data set), this is highly unlikely because New Zealand and the UK yielded a similar number of taxa indicative of deposited fine sediment (across all three analyses) despite the size of the UK data set being an order of magnitude greater than for New Zealand. It is more plausible that the rivers studied in Brazil generally contained naturally high levels of deposited fine sediment (the highest in our study, Figure S1 in Data S2) due to most rivers in the Cerrado Biome flowing over highly erodible sedimentary rocks (Macedo et al., 2014). As such, it is highly likely the community was pre‐adapted to higher fine sediment loads and therefore did not exhibit abrupt changes in taxonomic composition across the gradient. Indeed, Brazil supported the lowest EPT index value of all countries, confirming the presence of a community dominated by taxa more tolerant of fine sediment in turn driving the higher identified threshold.

Excessive fine sediment deposition is of particular concern in lowland gravel‐bed rivers where relatively stable seasonal flow regimes (often exacerbated by groundwater abstraction), coupled with an increase in arable farming, has resulted in these rivers being most at risk of fine sediment accumulation (Naden et al., 2016). Meanwhile, coarse‐bed rivers dominated by run‐off in upland sites maintain naturally lower levels of fine sediment. In our study, invertebrate communities in the UK showed some resilience to deposited fine sediment and most significant taxonomic changes occurred in the range of 30%–50% cover. However, this result should be interpreted with some caution, as these threshold values could exceed the tolerances of some communities and the implications of fine sediment deposition are likely to be context specific (Mathers et al., 2022). Sampling sites in all countries were biased towards lowland areas (relative to the number of upland sites sampled; Table S2 in Data S1), and weaker ecological responses to deposited fine sediment in lowland rivers have been recorded at both the community (Mathers et al., 2022; Matthaei et al., 2006) and the species level (Conroy et al., 2018). This suggests both environmental filtering of taxa in lowland areas and potentially some plasticity in response to deposited fine sediment. Thus, lowland species may be naturally less sensitive due to their continued exposure. Therefore, the thresholds identified in our study may potentially be too high for ecologically important taxa found in upland rivers globally. It is therefore likely that a context‐specific approach to fine sediment thresholds is needed and further assessment encompassing a range of river typologies is required (e.g. as in New Zealand's policy; Ministry for the Environment, 2020). To address the threat of excessive fine sediment deposition, field and experimental studies are needed that span a range of stream typologies (encompassing different geologies and flow regimes) and depositional/erosional sedimentation processes (saltation, suspension, bedload and clogging), to allow the influence of abiotic and biotic factors to be evaluated. This approach may warrant cross‐country/eco‐region collaboration as has been successfully undertaken for other ecosystem processes (e.g., Chauvet et al., 2016).

In most cases, the functional trait thresholds for deposited fine sediment were higher than the taxonomic thresholds. This result likely highlights the extent of functional redundancy, whereby other persistent taxa fulfil similar functional roles to taxa that are lost at low values of the gradient (Oliver et al., 2015). When considering thresholds by response group identity (i.e., *z*− and *z*+), taxa responding positively generally demonstrated a higher threshold than taxa responding negatively, whilst the opposite was true for traits. A similar converse pattern in taxa and trait responses has been demonstrated for flow thresholds (Monk et al., 2018), which may represent an artefact of the fuzzy coding of traits typically employed, with positively responding taxa increasing in occurrence and abundance before negatively responding taxa are completely lost. As selection pressures do not act on single traits, but on organisms possessing many interacting traits (Verberk et al., 2013), this pattern may also represent associated traits increasing or decreasing in their frequency of occurrence without being directly affected by the stressor. Moreover, the wide confidence intervals recorded here in all geographical regions suggest that functional responses to fine sediment are equivocal, with trait categories potentially failing to capture the mechanisms behind sensitivity (Wilkes et al., 2017). This is further evidenced with the response of functional community metrics across all geographic regions being inconsistent, implying that we should not use these metrics as indicators of fine sediment pressure with confidence. Two individual functional traits, feeding mode of herbivore and respiration via gills, consistently responded negatively to fine sediment in all four countries. Both these traits (and many others) have been cited as demonstrating variable results in a number of studies (see Murphy et al., 2017; Wilkes et al., 2017 for summaries). However, our study is the first to incorporate a large spatial scale and a wide range of fine sediment cover in each country; thus, our findings potentially suggest these two traits could represent mechanistic response traits. When all taxonomic metrics were considered, indices based on EPT groups were consistently the strongest indicators of fine sediment pressure across all countries, supporting existing evidence of the efficacy of this metric in the absence of sediment‐specific indices (Conroy et al., 2016b; McKenzie et al., 2022a).

Our findings suggest that there are varying levels of correspondence between the taxonomic and functional biodiversity facets along the deposited fine sediment gradient. Given the possible explanation of landscape filtering effects for our findings, we conclude that understanding regional context and historical land management practices are important in determining the response of communities to fine sediment gradients (Chen & Olden, 2020; Firmiano, Canedo‐Argüelles, et al., 2021; Firmiano, Castro, et al., 2021; Mathers et al., 2022). The application of meaningful deposited fine sediment targets continues to draw scientific debate and recommendations for the development of any future guidelines should focus on the implementation of a holistic approach such as the inclusion of catchment drivers, sediment regimes and channel morphology, coupled with ecologically relevant responses (Collins et al., 2011; Mondon et al., 2021). The significance of fine sediment as a global stressor has repercussions beyond in‐stream ecological degradation, with management considerations interconnected with future development and food security policies. When coupled with the implications of climate change, excess fine sediment stress remains a topic that requires significant attention from academics and environment managers. Our findings provide an important step towards a global perspective of this important issue.

## AUTHOR CONTRIBUTIONS

**Morwenna McKenzie:** Conceptualization; data curation; formal analysis; investigation; methodology; visualization; writing – original draft; writing – review and editing. **Andrew Brooks:** Investigation; writing – review and editing. **Marcos Callisto:** Investigation; writing – review and editing. **Adrian L. Collins:** Investigation; writing – review and editing. **Jessica M. Durkota:** Investigation; writing – review and editing. **Russell G. Death:** Investigation; writing – review and editing. **J. Iwan Jones:** Investigation; writing – review and editing. **Marden S. Linares:** Investigation; writing – review and editing. **Christoph D. Matthaei:** Investigation; writing – review and editing. **Wendy A. Monk:** Writing – review and editing. **John F. Murphy:** Investigation; writing – review and editing. **Annika Wagenhoff:** Investigation; writing – review and editing. **Martin Wilkes:** Methodology; writing – review and editing. **Paul J. Wood:** Methodology; writing – review and editing. **Kate L. Mathers:** Conceptualization; funding acquisition; investigation; methodology; project administration; supervision; writing – original draft; writing – review and editing.

## CONFLICT OF INTEREST STATEMENT

The authors declare no conflict of interest.

## Supporting information


Data S1.

Data S2.

## Data Availability

The data that support the findings of this study are openly available in Loughborough University repository at http://doi.org/10.17028/rd.lboro.24615570.
