# Supplementary material for: Freshwater invertebrate responses to fine sediment stress: A multi‐continent perspective
Source: Glob Chang Biol. 2023 Dec 9;30(1):e17084. doi: 10.1111/gcb.17084 (PMC10952627; doi:10.1111/gcb.17084)
Supplement: Supplementary file 1 — Data S1. [file GCB-30-0-s001.docx]

**Supplementary 1. Source data summaries**

A total of 6491 samples were retained from all regions across a range of river types. Site maps of retained sites and densities of fine sediment gradients (as visual fine sediment %) can be found in Figure S.1.


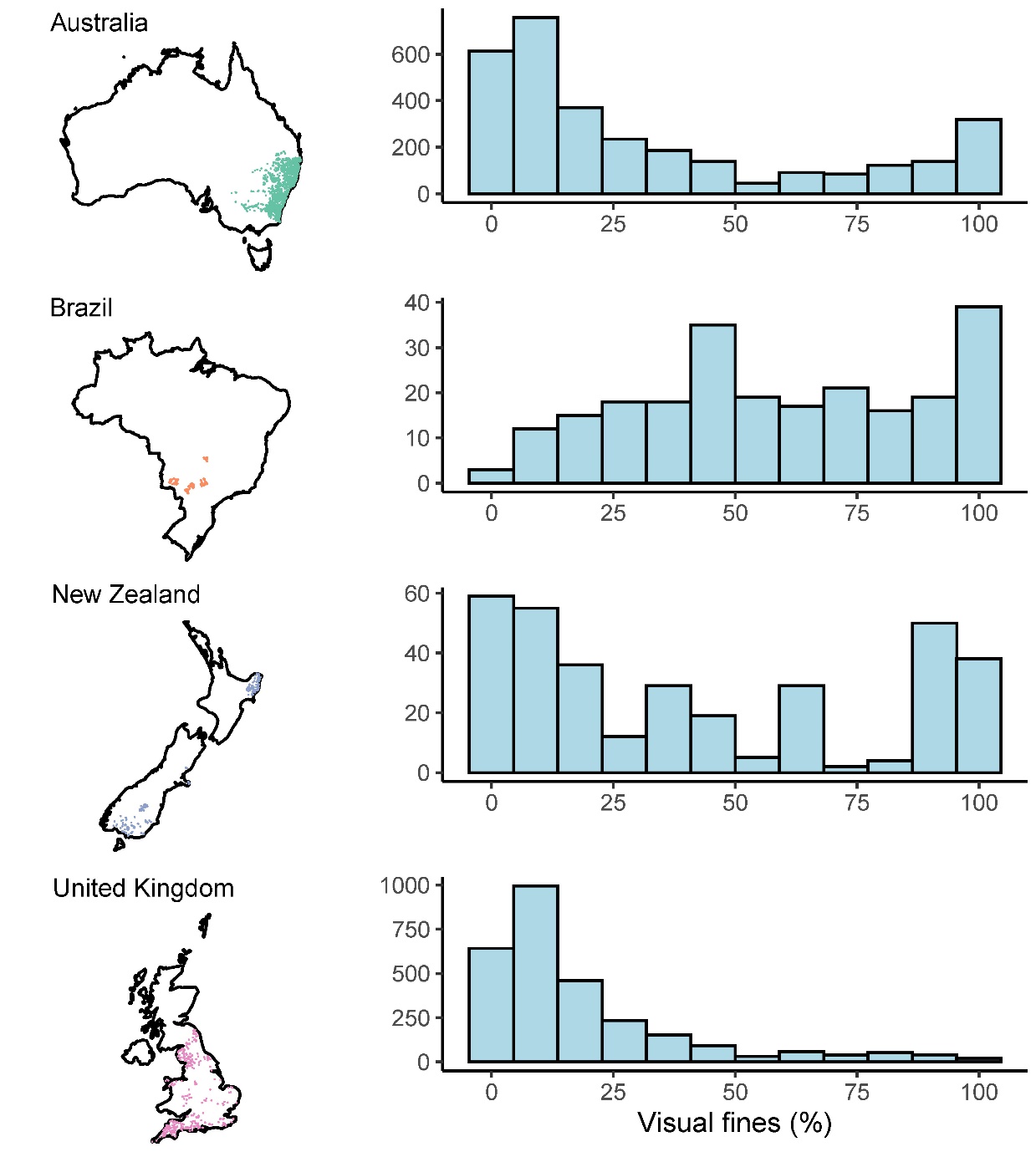


Figure S.1 Sampling locations and histograms of fine sediment gradient (as visual estimates %) for each region.

- 1. **Australia**

Data comprises an aggregation of records collected on behalf of the New South Wales (NSW) Government as part of the Monitoring River Health Project (1994 – 2000), the Sustainable Rivers Audit (2002 – 2012) and the Monitoring Evaluation and Reporting program (2006 – ongoing). Data is freely available online via the NSW Government data portal (<https://datasets.seed.nsw.gov.au/dataset/aquatic-macroinvertebrates-of-nsw-1994-ongoingc357e>). Following the AUSRIVAS protocol for assessment, only samples from spring (March 15 – June 15) and autumn (September 15 – December 15) were retained. Invertebrate data were collected and processed according to the Australia River Assessment System (AusRivAS) protocol (Smith et al., 1999; Turak et al., 2004), which ensures a standard methodology for collecting and processing biological (invertebrate) and environmental data. In summary, biological samples are collected via a D-net kick sample (250 µm mesh size). Sampling is undertaken separately at distinct riffle and edge habitats. Riffles are defined as habitats of broken water with rapid velocities and some cobble and boulder substratum and edge as those with little or no flow with organic matter and silt deposits. Both riffle and edge samples were retained as individual samples for analyses. Invertebrates are live-picked post collection following a standardised process to ensure consistent effort and performance and preserved (70% ethanol with 2% glycerol) prior to identification. Taxa were identified to family level with the exception of Oligochaeta (class), Polychatea (class), Ostracoda (subclass), Nematoda (phylum), Acarina (order) and Chironomidae (subfamily). Substrate was recorded at each habitat using visual estimates of the percentage cover of bedrock, boulder (>200 mm), cobble (60 – 200 mm), pebble (20 – 60 mm), gravel (2 – 20 mm), sand (0.02 – 2 mm), silt (0.002 – 0.02 mm) and clay (<0.002 mm).

In order to remove variability from co-occurring stressors, the AusRivAS scores for each habitat was used as a data filter. The AusRivAS system compares the observed community at a given site to that which would be expected to occur if the site was undisturbed or in reference condition. Only sites considered to pass the AusRivAS assessment were retained (this includes sites in bands “X” and “A”). Lastly, to reduce influence from large river types, sites from reaches classified as Strahler order >5 according to the HydroRIVERS geographical database were removed (Lehner et al., 2011).

**1.2 Brazil**

Data were collated from research projects conducted in the Cerrado biome (neotropical savanna), Brazil (Agra et al., 2021; Callisto et al., 2019; Castro et al., 2017, 2018, 2020; Firmiano et al., 2021; Macedo et al., 2018; D. R. O. Silva et al., 2018; L. F. R. Silva et al., 2021). Biological and physical data were collected according to the US-EPA protocols (USEPA - United States Environmental Protection Agency, 2016) adapted for use in neotropical savanna headwater streams (Callisto et al., 2014). The stream sites were selected using a probability-based procedure and a spatially balanced design (Macedo et al., 2014; Olsen & Peck, 2008).

Each sample reach was divided into 11 equally spaced transects and biological and environmental data collected at each transect. Invertebrates were collected in September, at the end of the dry period, using a kicknet (30 cm aperture, 500 µm mesh size) in a systematic zig-zag sequence across the 11 transects per site. The samples were aggregated to form one composite sample per site totalling ~1 m^2^, preserved in 4% formalin and processed in the laboratory. Taxa were identified to predominantly family level with EPT (Ephemeroptera, Plecoptera and Trichoptera) orders identified to genus level. Substrate estimates were also carried out following the US-EPA guidelines across the 11 transects at each site. Visual estimates of the substrate were categorized as percentages of bedrock (>4000 mm), boulder (>250-4000 mm), cobble (>64-250 mm), coarse gravel (>16-64 mm), fine gravel (>2-16 mm), sand (0.06-2 mm), and fines (silt and clay <0.06 mm). As data were collected for academic studies, sampling locations were selected to be relatively unaffected by confounding pressures. However, any samples that appeared as anomalies in terms of water quality (dissolved oxygen, total nitrates and total phosphates) were excluded.

**1.3 New Zealand**

Data for New Zealand comprised an aggregate of several academic studies. Data collected by Hunter (2020) comprised data from the North Island with the remaining datasets from the South Island. Ecological data was collected via the standard quantitative methods of kick and Surber samples (Table S.1). All samples were preserved in the field and subsequently identified to the lowest taxonomic resolution possible for most taxa groups (Table S.1). In the case of Magbanua et al., (2010), the original study comprised a before/after experimental design and for the purposes of this study, only the ‘before’ data was retained for analysis. Fine sediment visual estimates followed the method developed by Clapcott et al., (2011) which aims to reduce subjectivity when taking in-stream visual estimates. This involves an operator using an underwater viewing box (or a bathyscope) to estimate the % fine sediment (<2 mm) within the gridded area of the streambed observed within the viewing area. The protocol is carried out at several locations across a number of random transects (specified in Table S.1) and the average percentage of fines per reach calculated. As the protocol involves assessing the overall percentage of fines only, other substrate categories are not available for New Zealand data.

Table S.1. Summary of source data for New Zealand.

| **Source reference** | **Sampling date** | **Invertebrate sampling method** | **Invertebrate counting/ identification method** | **Fine sediment sampling protocol** |
| --- | --- | --- | --- | --- |
| (Hunter, 2020)  n = 206 | 2016 – 2018 (December – April) | D-net kick sample (x5 kick samples of 0.2 m^2^).  Preserved in 70% isopropyl alcohol | Fixed count of 200 plus scan for rare taxa. Identified to mixed taxon level (usually genus). | Bankside visual estimates of sampling reach |
| (Lange et al., 2014)  n = 43 | March – April 2011 | D-net kick sample.  Preserved in 70% ethanol. | Fixed count of 300 plus scan for rare taxa. Identified to genus level (except Diptera, Coleoptera and Crustacea to family level) | Visual estimates at 10 random locations using a viewing box (area 12 x 12 cm) |
| (Magbanua et al., 2010)  n = 36 | February 2007 | Surber samples (x 3).  Preserved in 90% ethanol. | Full count (plus subsampling for very abundant taxa and large samples). | Visual estimates at two random locations in each reach using a viewing box (diameter 20 cm) |
| (Matthaei et al., 2006)  n = 24 | January 2003 | Surber samples (x 4). Preserved in 70% ethanol. | Full count (plus subsampling for very abundant taxa). Identified to lowest taxonomic level possible. | Visual estimates at 20 random locations using a viewing box (area 25 x 25 cm) |
| (Wagenhoff et al., 2011)  n = 43 | January 2008 | D-net kick sample.  Preserved in ethanol. | Fixed count of 200 plus scan for rare taxa. | Visual estimates at two random locations across five transects using a viewing box (area 12 x 12 cm) |

**1.4 United Kingdom**

The Environment Agency, the regulatory authority in England, routinely collects invertebrate data for monitoring purposes and biological data is openly available online via the Ecology and Fish Data Explorer (<https://environment.data.gov.uk/ecology/explorer/>). Sample sites were screened to retain those that fulfilled the following criteria: (a) upstream catchment area greater than 10 km^2^, (b) located near a water quality monitoring site that was not downstream of discharge from a sewage treatment plant, (c) diffuse sources accounted for at least 70% of the total pollutant load on sampled days; (d) physicochemical parameters (pH, temperature, ammonia, phosphate and dissolved oxygen) were not classified as ‘Poor’ and ‘Bad’ according to Water Framework Directive (WFD) (2000/60/EC) classification; and (e) ecological parameters (invertebrates) were not classified as ‘Moderate’, ‘Poor’ or ‘Bad’.

Only data collected in either spring (March - May) or autumn (September - November) were retained. The Environment Agency follows standard protocols for sampling, processing, and identification of invertebrate samples. Samples were collected via the standard multi-habitat 3-minute kick survey (1 mm mesh size) followed by a 1-minute hand search for surface dwelling or animals attached to logs, stones, overhanging vegetation or other solid objects (Environment Agency, 2014b). Sampling time was distributed proportionally between the habitats present in the sampling area (e.g., riffle, marginal, pool etc). Following collection, the contents of the pond net were preserved (100% Industrial Methylated Spirits) and samples processed following Environment Agency operational instructions for the sorting and identification of invertebrates in the laboratory (Environment Agency, 2014a). Invertebrates were identified to mixed taxon level with most insect taxa identified to species level. Visual estimates of the substrate composition were carried out using the River Invertebrate Classification System (RIVPACS) categories: boulders (>256 mm), cobbles (64 – 256 mm), pebbles (16 – 64 mm), gravel (2 – 16 mm), sand (0.06 – 2 mm), silt (<0.06 mm), and clay (<0.06 mm sticky and cohesive).

A second data source collected for academic research (Murphy et al., 2015, 2017) was also collated that included additional sites from rural catchments in England and Wales. Sample sites were selected following filtering criterion applied to River Habitat Survey (RHS) stream sites (Environment Agency, 2003) using a combination of RHS data and GIS models (e.g. PSYCHIC) (Naura et al., 2016; Strömqvist et al., 2008). Sites were retained that were subject to fine sediment stress from agricultural sources (>75% as a proportion of total sediment input) and were not affected by confounding disturbance pressures (e.g., downstream of sewage treatment works, lakes and reservoirs, or urban areas). Following filtering, the final site list was selected to ensure a range of river types and sediment pressures were incorporated. Additional data from headwater streams in Wales (Jones et al., 2017) were provided from sites located in independent watersheds in agricultural catchments with low urban/suburban (<10%) and forestry (<10%) cover. All sites in the UK were sampled in spring and autumn. As with the Environment Agency data, invertebrates were sampled using a standard 3-minute kick (1 mm mesh size) followed by a 1-minute hand search. Habitats were sampled proportional to their total coverage within the reach. Invertebrates were fixed (10% formalin) and identified in a laboratory to mixed taxon level.

**1.5 Regional environmental variation**

We used the RiverATLAS (Linke et al., 2019) in the HydroATLAS database to obtain the following environmental variables for each region: long-term (1971-2000) average ‘naturalized’ discharge (m^3^ s^-1^) according to the global integrated water model WaterGap v2.2 (Döll et al., 2003), degree of flow regulation expressed as the percent ratio (%) between the total reservoir storage volume of all dams or impoundments on or upstream of the reach and the total annual discharge volume (Lehner et al., 2011), elevation in reach catchment (m) and stream gradient (decimeters per km) according to EarthEnv-DEM90 digital elevation model (Robinson et al., 2014), air temperature (°C) in reach catchment according to WorldClim v1.4 (Hijmans et al., 2005), percentage (%) of reach catchment used for agricultural land (and the constituent elements of cropland and pasture %) according to EarthStat global dataset (Ramankutty et al., 2008), and urban extent (%) in reach catchment according to the Global Human Settlement Framework (Pesaresi et al., 2016).

Table S.2 Mean and standard deviation (SD) values of environmental variables for each dataset, acquired from RiverATLAS (<https://www.hydrosheds.org/page/hydroatlas>).

|  | **Australia** | **Brazil** | **United Kingdom** | **New Zealand** |
| --- | --- | --- | --- | --- |
| **Natural discharge (m^3^/s)** | 2.8 (7.4) | 44.5 (482.3) | 0.9 (1.1) | 5.4 (13.3) |
| **Flow regulation (%)** | 12.9 (65.4) | 0.6 (7.0) | 0.9 (15.1) | 0.1 (0.8) |
| **Elevation (m)** | 500.6 (382.1) | 394.2 (110.7) | 185.2 (110.9) | 320.4 (233.7) |
| **Stream gradient (decimeters per km)** | 94.3 (141.6) | 41.8 (31.4) | 102.2 (88.0) | 106.9 (109.4) |
| **Air temperature (°C)** | 15.1 (2.9) | 23.0 (0.8) | 8.8 (1.0) | 10.7 (2.1) |
| **Agricultural land (%)**  **As cropland (%)**  **As pasture (%)** | 31.4 (34.6) | 81.7 (16.3) | 66.9 (19.8) | 44.8 (32.5) |
|  | 7.3 (16.1) | 10.7 (9.5) | 26.0 (20.7) | 4.2 (8.4) |
|  | 24.0 (28.7) | 71.0 (18.2) | 40.9 (18.4) | 40.6 (30.3) |
| **Urban land (%)** | 0.9 (5.8) | 0.8 (5.5) | 2.4 (8.0) | 1.0 (7.5) |

Table S.3 Trait categories and modalities used in analyses (Kunz et al., 2022)

| **Trait category** | **Modes** | **Short name** |
| --- | --- | --- |
| Voltinism (generations per year) | <1  1  >1 | vol_semi  vol_uni  vol_multi |
| Oviposition | Aquatic eggs  Ovoviviparity  Terrestrial eggs | ovip_aqu  ovip_ovo  ovip_ter |
| Locomotion and relationship to substrate | Burrower  Crawler  Sessile  Swimmer | locom_burrow  locom_crawl  locom_sessi  locom_swim |
| Body form | Cylindrical  Flattened  Spherical  Streamlined | bf_cylindrical  bf_flattened  bf_spherical  bf_streamlined |
| Feeding mode | Filterer  Gatherer  Herbivore  Parasite  Predator  Shredder | feed_filter  feed_gatherer  feed_herbivore  feed_parasite  feed_predator  feed_shredder |
| Respiration | Gills  Plastron/spiracle  Tegument | resp_gil  resp_pls_spi  resp_teg |

**Acknowledgements**

MC and MSL were supported by the Fundação de Amparo à Pesquisa do Estado de Minas Gerais – FAPEMIG (PPM-00104-18 to MC), Conselho Nacional de Desenvolvimento Científico e Tecnológico (CNPq, research productivity grants 304060/2020-8 to MC), Coordenação de Aperfeiçoamento de Pessoal de Nível Superior (CAPES) – Finance Code 001. MSL received a post-doc scholarship from ANEEL/CEMIG (P&D GT-599; Project PROECOS). The contribution of ALC to this paper was funded by the UKRI-BBSRC (Biotechnology and Biological Sciences Research Council) institute strategic programme Resilient Farming Futures. JIJ, ALC and JFM were supported by U.K. Government Department for Environment, Food and Rural Affairs (Defra project WQ0128) and the Welsh Government Agri-environment monitoring and technical services Contract. Lot 3: soil, water and climate change (Ecosystems) No. 183/2007/08.

**References**

Agra, J., Ligeiro, R., Heino, J., Macedo, D. ., Castro, D. M. ., Linares, M. ., & Callisto, M. (2021). Anthropogenic disturbances alter the relationships between environmental heterogeneity and biodiversity of stream insects. *Ecological Indicators*, *121*, 107079. https://doi.org/10.1016/J.ECOLIND.2020.107079

Callisto, M., Hughes, R. M., Lopes, J. M., & Castro, M. A. (2014). *Ecological conditions in hydropower basins* (3rd ed). Companhia Energetica de Minas Gerais. https://scholar.google.com/scholar?hl=en&as_sdt=0%2C5&q=Ecological+conditions+in+hydropower+basins+%283rd+ed.%29.&btnG=#d=gs_cit&t=1651663517831&u=%2Fscholar%3Fq%3Dinfo%3Akk--m70GxW8J%3Ascholar.google.com%2F%26output%3Dcite%26scirp%3D0%26hl%3Den

Callisto, M., Macedo, D. R., Linares, M. S., & Hughes, R. M. (2019). Multi-status and multi-spatial scale assessment of landscape effects on benthic macroinvertebrates in the Neotropical Savanna. In R. M. Hughes, D. M. Infante, L. Wang, K. Chen, & B. F. Terra (Eds.), *Advances in Understanding Landscape Influences on Freshwater Habitats and Biological Assemblages* (pp. 275–302). American Fisheries Society Symposium 90.

Castro, D. M. ., Dolédec, S., & Callisto, M. (2017). Landscape variables influence taxonomic and trait composition of insect assemblages in Neotropical savanna streams. *Freshwater Biology*, *62*(8), 1472–1486. https://doi.org/10.1111/FWB.12961

Castro, D. M. ., Dolédec, S., & Callisto, M. (2018). Land cover disturbance homogenizes aquatic insect functional structure in neotropical savanna streams. *Ecological Indicators*, *84*, 573–582. https://doi.org/10.1016/J.ECOLIND.2017.09.030

Castro, D. M. P., da Silva, P. G., Solar, R., & Callisto, M. (2020). Unveiling patterns of taxonomic and functional diversities of stream insects across four spatial scales in the neotropical savanna. *Ecological Indicators*, *118*, 106769. https://doi.org/10.1016/J.ECOLIND.2020.106769

Clapcott, J. E., Young, R. G., Harding, J. S., Matthaei, C. D., Quinn, J. M., & Death, R. G. (2011). *Sediment assessment methods: protocols and guidelines for assessing the effects of deposited fine sediment on in-stream values*.

Döll, P., Kaspar, F., & Lehner, B. (2003). A global hydrological model for deriving water availability indicators: model tuning and validation. *Journal of Hydrology*, *270*(1–2), 105–134. https://doi.org/10.1016/S0022-1694(02)00283-4

Directive 2000/60/EC of the European Parliament and of the Council Establishing a Framework for the Community Action in the Field of Water Policy., L327 The European Parliament and the Council of the European Union: Brussels, Belgium. 1 (2000). https://doi.org/10.1039/ap9842100196

Environment Agency. (2003). *River Habitat Survey in Britain and Ireland: FieldSurvey Guidance Manual River Habitat Survey Manual: 2003 Version*.

Environment Agency. (2014a). *Freshwater macro-invertebrate analysis of riverine samples. Operational instruction 024_08*.

Environment Agency. (2014b). *Freshwater macro-invertebrate sampling in rivers. Operational instruction 018_08.*

Firmiano, K. R., Castro, D. M. P., Linares, M. S., & Callisto, M. (2021). Functional responses of aquatic invertebrates to anthropogenic stressors in riparian zones of Neotropical savanna streams. *Science of The Total Environment*, *753*, 141865. https://doi.org/10.1016/J.SCITOTENV.2020.141865

Hijmans, R. J., Cameron, S. E., Parra, J. L., Jones, P. G., & Jarvis, A. (2005). Very high resolution interpolated climate surfaces for global land areas. *International Journal of Climatology*, *25*(15), 1965–1978. https://doi.org/10.1002/JOC.1276

Hunter, H. (2020). *THE EFFECT OF LANDUSE AND GEOLOGY ON MACROINVERTERBATE COMMUNITIES IN EAST COAST STREAMS, GISBORNE, NEW ZEALAND.* Massey University.

Jones, J. I., Murphy, J. F., Anthony, S. G., Arnold, A., Blackburn, J. H., Duerdoth, C. P., Hawczak, A., Hughes, G. O., Pretty, J. L., Scarlett, P. M., Gooday, R. D., Zhang, Y. S., Fawcett, L. E., Simpson, D., Turner, A. W. B., Naden, P. S., & Skates, J. (2017). Do agri-environment schemes result in improved water quality? *Journal of Applied Ecology*, *54*(2), 537–546. https://doi.org/10.1111/1365-2664.12780

Kunz, S., Kefford, B. J., Schmidt-Kloiber, A., Matthaei, C. D., Usseglio-Polatera, P., Graf, W., Poff, N. L. R., Metzeling, L., Twardochleb, L., Hawkins, C. P., & Schäfer, R. B. (2022). Tackling inconsistencies among freshwater invertebrate trait databases: harmonising across continents and aggregating taxonomic resolution. *Freshwater Biology*, *67*(2), 275–291. https://doi.org/10.1111/FWB.13840

Lange, K., Townsend, C. R., & Matthaei, C. D. (2014). Can biological traits of stream invertebrates help disentangle the effects of multiple stressors in an agricultural catchment? *Freshwater Biology*, *59*(12), 2431–2446. http://doi.wiley.com/10.1111/fwb.12437

Lehner, B., Liermann, C. R., Revenga, C., Vörömsmarty, C., Fekete, B., Crouzet, P., Döll, P., Endejan, M., Frenken, K., Magome, J., Nilsson, C., Robertson, J. C., Rödel, R., Sindorf, N., & Wisser, D. (2011). High-resolution mapping of the world’s reservoirs and dams for sustainable river-flow management. *Frontiers in Ecology and the Environment*, *9*(9), 494–502. https://doi.org/10.1890/100125

Linke, S., Lehner, B., Ouellet Dallaire, C., Ariwi, J., Grill, G., Anand, M., Beames, P., Burchard-Levine, V., Maxwell, S., Moidu, H., Tan, F., & Thieme, M. (2019). Global hydro-environmental sub-basin and river reach characteristics at high spatial resolution. *Scientific Data 2019 6:1*, *6*(1), 1–15. https://doi.org/10.1038/s41597-019-0300-6

Macedo, D. ., Pompeu, P. ., Morais, L., Castro, M. ., Alves, C. B. M., França, J. S., Sanches, B. ., Agra, J. U. ., & Callisto, M. (2014). Sampling site selection, land use and cover, field reconnaissance, and sampling. In M. Callisto, R. M. Hughes, J. M. Lopes, & M. A. Castro (Eds.), *Ecological conditions in hydropower basins. Serie Peixe Vivo 3.* (pp. 61–83). Companhia Energética de Minas Gerais.

Macedo, D. R., Hughes, R. M., Kaufmann, P. R., & Callisto, M. (2018). Development and validation of an environmental fragility index (EFI) for the neotropical savannah biome. *Science of The Total Environment*, *635*, 1267–1279. https://doi.org/10.1016/J.SCITOTENV.2018.04.216

Magbanua, F. S., Townsend, C. R., Blackwell, G. L., Phillips, N., & Matthaei, C. D. (2010). Responses of stream macroinvertebrates and ecosystem function to conventional, integrated and organic farming. *Journal of Applied Ecology*, *47*(5), 1014–1025. https://doi.org/10.1111/J.1365-2664.2010.01859.X

Matthaei, C. D., Weller, F., Kelly, D. W., & Townsend, C. R. (2006). Impacts of fine sediment addition to tussock, pasture, dairy and deer farming streams in New Zealand. *Freshwater Biology*, *51*(11), 2154–2172. https://doi.org/10.1111/j.1365-2427.2006.01643.x

Murphy, J. F., Jones, J. I., Arnold, A., Duerdoth, C. P., Pretty, J. L., Naden, P. S., Sear, D. A., & Collins, A. L. (2017). Can macroinvertebrate biological traits indicate fine-grained sediment conditions in streams? *River Research and Applications*, *33*(10), 1606–1617. https://doi.org/10.1002/rra.3194

Murphy, J. F., Jones, J. I., Pretty, J. L., Duerdoth, C. P., Hawczak, A., Arnold, A., Blackburn, J. H., Naden, P. S., Old, G., Sear, D. A., Hornby, D., Clarke, R. T., & Collins, A. L. (2015). Development of a biotic index using stream macroinvertebrates to assess stress from deposited fine sediment. *Freshwater Biology*, *60*(10), 2019–2036. https://doi.org/10.1111/fwb.12627

Naura, M., Hornby, D. D., Collins, A. L., Sear, D. A., Hill, C., Jones, J. I., & Naden, P. S. (2016). Mapping the combined risk of agricultural fine sediment input and accumulation for riverine ecosystems across England and Wales. *Ecological Indicators*, *70*, 209–221. https://doi.org/10.1016/J.ECOLIND.2016.03.055

Olsen, A. R., & Peck, D. V. (2008). Survey design and extent estimates for the Wadeable Streams Assessment. *Journal of the North American Benthological Society*, *27*(4), 822–836. https://doi.org/10.1899/08-050.1

Pesaresi, M., Melchiorri, M., Siragusa, A., & Kemper, T. (2016). *Atlas of the Human Planet - Mapping Human Presence on Earth with the Global Human Settlement Layer. EUR 28116.* https://doi.org/10.2788/582834

Ramankutty, N., Evan, A. T., Monfreda, C., & Foley, J. A. (2008). Farming the planet: 1. Geographic distribution of global agricultural lands in the year 2000. *Global Biogeochemical Cycles*, *22*(1), 1003. https://doi.org/10.1029/2007GB002952

Robinson, N., Regetz, J., & Guralnick, R. P. (2014). EarthEnv-DEM90: A nearly-global, void-free, multi-scale smoothed, 90m digital elevation model from fused ASTER and SRTM data. *ISPRS Journal of Photogrammetry and Remote Sensing*, *87*, 57–67. https://doi.org/10.1016/J.ISPRSJPRS.2013.11.002

Silva, D. R. O., Herlihy, A. ., Hughes, R. ., Macedo, D. ., & Callisto, M. (2018). Assessing the extent and relative risk of aquatic stressors on stream macroinvertebrate assemblages in the neotropical savanna. *Science of The Total Environment*, *633*, 179–188. https://doi.org/10.1016/J.SCITOTENV.2018.03.127

Silva, L. F. R., Castro, D. M. ., Juen, L., Callisto, M., Hughes, R. ., & Hermes, M. . (2021). Functional responses of Odonata larvae to human disturbances in neotropical savanna headwater streams. *Ecological Indicators*, *133*, 108367. https://doi.org/10.1016/J.ECOLIND.2021.108367

Smith, M. J., Kay, W. R., Edward, D. H. D., Papas, P. J., Richardson, K. S. J., Simpson, J. C., Pinder, A. M., Cale, D. J., Horwitz, P. H. J., Davis, J. A., Yung, F. H., Norris, R. H., & Halse, S. A. (1999). AusRivAS: using macroinvertebrates to assess ecological condition of rivers in Western Australia. *Freshwater Biology*, *41*(2), 269–282. https://doi.org/10.1046/J.1365-2427.1999.00430.X

Strömqvist, J., Collins, A. L., Davison, P. S., & Lord, E. I. (2008). PSYCHIC – A process-based model of phosphorus and sediment transfers within agricultural catchments. Part 2. A preliminary evaluation. *Journal of Hydrology*, *350*(3–4), 303–316. https://doi.org/10.1016/J.JHYDROL.2007.10.044

Turak, E., Waddel, N., & Johnstone, G. (2004). *New South Wales (NSW) Australian River Assessment System (AUSRIVAS) Sampling and Processing Manual 2004*.

USEPA - United States Environmental Protection Agency. (2016). *National Rivers and Streams Assessment 2013-2014: Field Operations Manual – Wadeable*.

Wagenhoff, A., Townsend, C. R., Phillips, N., & Matthaei, C. D. (2011). Subsidy-stress and multiple-stressor effects along gradients of deposited fine sediment and dissolved nutrients in a regional set of streams and rivers. *Freshwater Biology*, *56*(9), 1916–1936. https://doi.org/10.1111/j.1365-2427.2011.02619.x
