# Supplementary material for: Freshwater invertebrate responses to fine sediment stress: A multi‐continent perspective
Source: Glob Chang Biol. 2023 Dec 9;30(1):e17084. doi: 10.1111/gcb.17084 (PMC10952627; doi:10.1111/gcb.17084)
Supplement: Supplementary file 2 — Data S2. [file GCB-30-0-s002.docx]

# **Supplementary 2. Supplementary results**

Table S.1 Threshold values of the percentage of deposited fine sediment for invertebrate communities identified by Gradient Forest. Threshold values defined as locations where compositional change is higher compared to the turnover occurring elsewhere across the gradient. The largest (i.e. the point at which the ratio of densities is largest) turnover for each individual model is indicated in bold.

|  | **Taxonomic** | **Functional** |
| --- | --- | --- |
| **Australia** | **11%,** 68%, 85%, 88%, 98% | **12%,** 23%, 36% |
| **Brazil** | **9%,** 22**%**, 44**%** | 44**%**, 85**%**, **94%** |
| **New Zealand** | 3%, 62**%**, **82%,** 98**%** | 39**%**, 50**%**, 56**%**, 62**%**, **89%** |
| **UK** | 16**%**, 22**%**, 30**%**, **44%,** 75%, 89%, 99% | 39**%**, 48%, **59%,** 65%, 74%, 90%, 99% |

Table S.2 Pairwise correlation between taxonomic indices and visual fine sediment %. Correlations have been corrected for multiple comparisons using the Holm-Bonferoni correction (Holm, 1979). *p* values are reported in brackets.

|  | **Australia** | **Brazil** | **UK** | **New Zealand** |
| --- | --- | --- | --- | --- |
| FRic | -**0.21** (<0.01) | -0.17 (>0.05) | **0.08** (<0.01) | 0.09 (>0.05) |
| FDis | **-0.13** (<0.01) | 0.05 (>0.05) | -0.04 (>0.05) | **0.23** (<0.01) |
| FDiv | **0.24** (<0.01) | -0.20 (>0.05) | -0.03 (>0.05) | **0.25** (<0.01) |
| FEve | **0.15** (<0.01) | 0.10 (>0.05) | -0.04 (>0.05) | **0.26** (<0.01) |
| RaoQ | **-0.20** (<0.01) | 0.04 (>0.05) | 0.00 (>0.05) | **0.23** (<0.01) |
| Taxa richness | **-0.09** (<0.01) | **-0.28** (<0.01) | **0.19** (<0.01) | 0.09 (>0.05) |
| Simpson | 0.04 (>0.05) | **-0.25** (<0.01) | 0.00 (>0.05) | -0.09 (>0.05) |
| Pielou | **0.10** (<0.01) | -0.27 (>0.05) | **-0.10** (<0.01) | -0.11 (>0.05) |
| EPTabundance | **-0.48** (<0.01) | **-0.27** (0.01) | **-0.48** (<0.01) | **-0.11** (<0.01) |
| EPTindex | **-0.48** (<0.01) | **-0.16** (0.01) | **-0.45** (<0.01) | **-0.11** (<0.01) |
| EPTrichness | **-0.58** (<0.01) | **-0.25** (<0.01) | **-0.22** (<0.01) | **-0.55** (<0.01) |


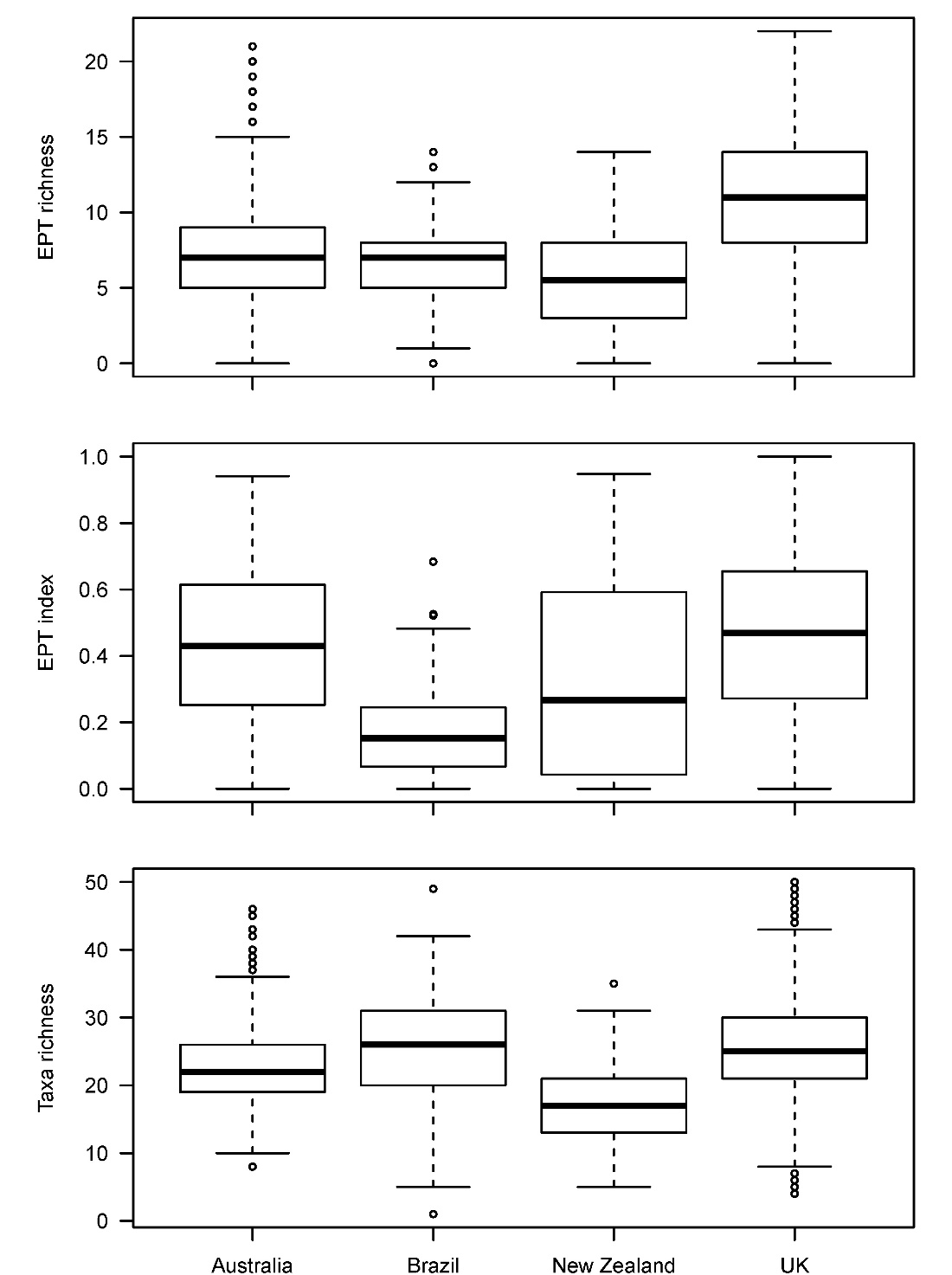


Figure S.1 Boxplots of EPT richness, EPT index and taxonomic richness for Australia, Brazil, UK, and New Zealand.

Table S.3 Full taxa identified by gradient forest analysis for which fine sediment (%) has predictive power on the composition across the gradient. Values represent R^2^.

| **Taxa** | **GF** | **Taxa** | **GF** |
| --- | --- | --- | --- |
| ***Australia*** | | | |
| Elmidae | 0.135 | Physidae | 0.025 |
| Hydropsychidae | 0.132 | Cladocera | 0.025 |
| Corydalidae | 0.129 | Conoesucidae | 0.024 |
| Philopotamidae | 0.101 | Diphlebiidae | 0.022 |
| Copepoda | 0.092 | Notonectidae | 0.021 |
| Psephenidae | 0.084 | Baetidae | 0.019 |
| Hydraenidae | 0.082 | Hydrophilidae | 0.017 |
| Hydrobiosidae | 0.081 | Ptilodactylidae | 0.015 |
| Dytiscidae | 0.080 | Ameletopsidae | 0.015 |
| Corixidae | 0.071 | Hydrometridae | 0.015 |
| Coenagrionidae | 0.051 | Atyidae | 0.013 |
| Parastacidae | 0.049 | Chironomidae | 0.012 |
| Palaemonidae | 0.048 | Mesoveliidae | 0.012 |
| Gripopterygidae | 0.047 | Aeshnidae | 0.011 |
| Calocidae | 0.040 | Blephariceridae | 0.010 |
| Eustheniidae | 0.038 | Ancylidae | 0.009 |
| Leptophlebiidae | 0.038 | Empididae | 0.008 |
| Athericidae | 0.038 | Naucoridae | 0.007 |
| Tipulidae | 0.031 | Neurorthidae | 0.007 |
| Veliidae | 0.031 | Dixidae | 0.007 |
| Simuliidae | 0.029 | Ephemerellidae | 0.005 |
| Hydrochidae | 0.029 | Gordiidae | 0.004 |
| Leptoceridae | 0.029 | Tasimiidae | 0.004 |
| Ostracoda | 0.028 | Caenidae | 0.003 |
| Coloburiscidae | 0.027 | Curculionidae | 0.001 |
| Glossosomatidae | 0.025 | Spercheidae | <0.001 |
| Ceratopogonidae | 0.025 |  |  |
| ***Brazil*** | | | |
| Megapodagrionidae | 0.019 | Psychodidae | 0.008 |
| ***New Zealand*** | | | |
| Phreatoicidae | 0.286 | Corydalidae | 0.029 |
| Leptophlebiidae | 0.209 | Dugesiidae | 0.023 |
| Oligochaeta | 0.203 | Hydrozoa | 0.021 |
| Nematoda | 0.177 | Saldidae | 0.017 |
| Tateidae | 0.106 | Ptilodactylidae | 0.015 |
| Coenagrionidae | 0.091 | Hydrobiosidae | 0.011 |
| Elmidae | 0.076 | Hydraenidae | 0.004 |
| Notonectidae | 0.068 | Tabanidae | 0.003 |
| Sphaeriidae | 0.062 | Corixidae | 0.002 |
| Dytiscidae | 0.050 | Sarcophagidae | 0.002 |
| Veliidae | 0.049 | Nesameletidae | 0.001 |
| Copepoda | 0.029 |  |  |
| ***UK*** | | | |
| Sphaeriidae | 0.173 | Nemouridae | 0.028 |
| Heptageniidae | 0.163 | Hydraenidae | 0.021 |
| Baetidae | 0.083 | Tateidae | 0.016 |
| Leuctridae | 0.073 | Perlidae | 0.015 |
| Rhyacophilidae | 0.063 | Physidae | 0.012 |
| Gammaridae | 0.052 | Goeridae | 0.003 |
| Perlodidae | 0.038 | Glossosomatidae | 0.001 |
| Hydropsychidae | 0.036 | Scirtidae | 0.001 |
| Chloroperlidae | 0.029 | Polycentropodidae | 0.001 |

Table S.4 Full traits identified by gradient forest analysis for which fine sediment (%) has predictive power on the composition across the gradient. Values represent R^2^. Trait abbreviations in Supplementary 1 Table S.3.

| **Traits** | **GF** | **Traits** | **GF** |
| --- | --- | --- | --- |
| ***Australia*** | | | |
| resp_pls_spi | 0.241 | feed_herbivore | 0.118 |
| locom_burrow | 0.215 | volt_bi_multi | 0.117 |
| locom_sessil | 0.209 | ovip_aqu | 0.114 |
| resp_gil | 0.193 | size_large | 0.109 |
| feed_predator | 0.186 | resp_teg | 0.071 |
| locom_swim | 0.167 | locom_crawl | 0.028 |
| feed_filter | 0.148 | feed_shredder | 0.025 |
| volt_uni | 0.129 | size_medium | 0.020 |
| ovip_ter | 0.121 | size_small | 0.004 |
| ***Brazil*** | | | |
| locom_burrow | 0.024 |  |  |
| ***New Zealand*** | | | |
| volt_bi_multi | 0.257 | resp_teg | 0.127 |
| volt_uni | 0.253 | feed_herbivore | 0.106 |
| feed_predator | 0.224 | resp_gil | 0.058 |
| locom_swim | 0.213 | size_small | 0.049 |
| resp_pls_spi | 0.183 | size_medium | 0.017 |
| ***UK*** | | | |
| size_large | 0.158 | feed_herbivore | 0.045 |
| size_medium | 0.087 | resp_pls_spi | 0.025 |
| ovip_aqu | 0.069 | feed_gatherer | 0.024 |
| ovip_ovo | 0.063 | locom_swim | 0.012 |
| size_small | 0.056 | volt_semi | 0.010 |
|  |  |  |  |

Table S.5 Full Threshold Indicator Analysis (TITAN) results for Australian taxa data. Results parameters include: zenv.cp which represents the environmental change point for each taxon, IndVal statistic from Dufrêne & Legendre (1997), IndVal zscore, 5% and 95% change point quantiles among bootstrap replicates, and filter which indicates the group assignment (i.e. either responding negatively (-ve) or positively (+ve)) for each taxon which met purity and reliability criteria. Only pure and reliable taxa are presented herein (where ≥95% of 999 bootstrap runs are significantly different from a random distribution where *p* <0.05).

|  | **zenv.cp** | **IndVal** | **zscore** | **5%** | **95%** | **filter** |
| --- | --- | --- | --- | --- | --- | --- |
| Aeshnidae | 10 | 32.88 | 24.06 | 10 | 20 | -ve |
| Ameletopsidae | 5 | 8.4 | 24.56 | 5 | 10 | -ve |
| Ancylidae | 90 | 18.96 | 16.97 | 60 | 98.025 | +ve |
| Antipodoecidae | 5 | 1.16 | 7.45 | 0 | 15.5 | -ve |
| Araneae | 60 | 4.74 | 9.98 | 15 | 90 | +ve |
| Argiolestidae | 10 | 9.74 | 10.55 | 5 | 35 | +ve |
| Athericidae | 5 | 16.47 | 34.67 | 5 | 15 | -ve |
| Atyidae | 10 | 43.8 | 20.52 | 10 | 25 | +ve |
| Austroperlidae | 5 | 7.05 | 13.96 | 5 | 31.525 | -ve |
| Baetidae | 10 | 53.6 | 19.99 | 5 | 20 | -ve |
| Belostomatidae | 30 | 2.76 | 13.05 | 20 | 100 | +ve |
| Blephariceridae | 0 | 5.08 | 21.63 | 0 | 5 | -ve |
| Caenidae | 20 | 41.37 | 14.97 | 15 | 30 | +ve |
| Calamoceratidae | 65 | 26.2 | 12.64 | 50 | 70 | -ve |
| Calocidae | 5 | 28.42 | 39.5 | 5 | 15 | -ve |
| Carabidae | 95 | 4.81 | 12.73 | 29.775 | 100 | +ve |
| Ceinidae | 5 | 6.71 | 8.25 | 5 | 25 | +ve |
| Ceratopogonidae | 65 | 35.96 | 20.63 | 40 | 90 | +ve |
| Chironomidae | 64.5 | 58.43 | 15.52 | 50 | 80 | +ve |
| Chrysomelidae | 100 | 8.17 | 12.97 | 11.65 | 100 | +ve |
| Cladocera | 95 | 30.38 | 34.91 | 60 | 100 | +ve |
| Coenagrionidae | 30 | 39.91 | 45.39 | 20 | 45 | +ve |
| Collembola | 30 | 10.79 | 12.88 | 15 | 30 | +ve |
| Coloburiscidae | 15 | 14.27 | 28.77 | 5 | 20 | -ve |
| Conoesucidae | 10 | 29.17 | 31.78 | 5 | 30 | -ve |
| Copepoda | 75 | 30.52 | 45.45 | 55 | 90 | +ve |
| Corallanidae | 90 | 6.9 | 24.18 | 65 | 95 | +ve |
| Corbiculidae | 60 | 10.86 | 5.76 | 18 | 95 | -ve |
| Corduliidae | 10 | 23.45 | 18.22 | 10 | 35 | +ve |
| Corixidae | 15 | 60.83 | 43.5 | 10 | 20 | +ve |
| Corydalidae | 15 | 44.55 | 59.74 | 5 | 15 | -ve |
| Culicidae | 80 | 18.95 | 20.03 | 20 | 95 | +ve |
| Curculionidae | 54.5 | 2.78 | 11.54 | 55 | 100 | +ve |
| Diphlebiidae | 10 | 9.4 | 28.11 | 2 | 15 | -ve |
| Diplopoda | 15 | 0.87 | 4.13 | 5 | 100 | +ve |
| Diptera | 75 | 7 | 4.48 | 39 | 95 | -ve |
| Dixidae | 80 | 17.93 | 10.69 | 50 | 90 | -ve |
| Dugesiidae | 4.5 | 15.76 | 5.94 | 0 | 25 | -ve |
| Dytiscidae | 16 | 56.95 | 47.15 | 10 | 40 | +ve |
| Ecnomidae | 2 | 16.08 | 3.37 | 0 | 75.25 | +ve |
| Elmidae | 10 | 64.83 | 56.9 | 10 | 20 | -ve |
| Empididae | 20 | 5.66 | 16.63 | 5 | 25 | -ve |
| Ephemerellidae | 5 | 2.2 | 13.26 | 5 | 10 | -ve |
| Eusiridae | 80 | 4.19 | 5.13 | 30 | 94.525 | -ve |
| Eustheniidae | 5 | 20.03 | 38.09 | 5 | 15 | -ve |
| Gerridae | 8 | 24.12 | 11.31 | 2 | 15 | +ve |
| Glossiphoniidae | 40 | 8.97 | 7.94 | 20 | 70 | +ve |
| Glossosomatidae | 5 | 18.97 | 33.98 | 5 | 10 | -ve |
| Gomphidae | 50 | 23.41 | 5.44 | 10 | 100 | -ve |
| Gordiidae | 8.5 | 6.95 | 18.25 | 5 | 15 | -ve |
| Gripopterygidae | 10 | 53.17 | 37.82 | 10 | 25 | -ve |
| Gyrinidae | 10 | 22.37 | 15.51 | 5 | 15 | +ve |
| Haliplidae | 20 | 3.83 | 13.21 | 20 | 96.1 | +ve |
| Hebridae | 30 | 3.29 | 8.45 | 5 | 98 | +ve |
| Helicophidae | 35 | 2.18 | 6.04 | 0 | 70 | -ve |
| Helicopsychidae | 20 | 21.43 | 28.3 | 8 | 20 | -ve |
| Heteroceridae | 90 | 1.49 | 11.64 | 80 | 100 | +ve |
| Hydraenidae | 40 | 38.72 | 41.43 | 30 | 80 | +ve |
| Hydrobiosidae | 10 | 50.83 | 54.9 | 5 | 16 | -ve |
| Hydrochidae | 60 | 11.46 | 29.56 | 60 | 75 | +ve |
| Hydrometridae | 30 | 16.38 | 25.2 | 20 | 85 | +ve |
| Hydrophilidae | 20 | 41.04 | 24.97 | 10 | 50 | +ve |
| Hydropsychidae | 5 | 60.37 | 57.07 | 5 | 15 | -ve |
| Hydroptilidae | 70 | 20.01 | 5.12 | 25 | 100 | -ve |
| Isostictidae | 10 | 8.1 | 13.43 | 10 | 90 | +ve |
| Leptoceridae | 5 | 58.6 | 23.7 | 1.475 | 10 | +ve |
| Leptophlebiidae | 70 | 63.57 | 29.41 | 47.85 | 80.25 | -ve |
| Lestidae | 50 | 8.4 | 16.68 | 25 | 60 | +ve |
| Lymnaeidae | 5 | 8.37 | 6.41 | 5 | 40 | +ve |
| Mesoveliidae | 50 | 10.73 | 25.07 | 30 | 90 | +ve |
| Naucoridae | 50 | 4.59 | 14.61 | 30 | 100 | +ve |
| Nepidae | 35 | 7.82 | 18.46 | 20 | 85 | +ve |
| Neurorthidae | 5 | 5.91 | 20.03 | 5 | 15 | -ve |
| Notonectidae | 29.5 | 48.21 | 41.72 | 15 | 35 | +ve |
| Notonemouridae | 80 | 7.35 | 8.03 | 35 | 79.025 | -ve |
| Ochteridae | 75 | 4.35 | 16.96 | 25 | 85 | +ve |
| Odontoceridae | 60 | 12.54 | 8.25 | 5 | 77.525 | -ve |
| Oligochaeta | 20 | 34.6 | 12.74 | 5 | 25 | -ve |
| Oniscidae | 80 | 2.01 | 7.35 | 15 | 100 | +ve |
| Oniscigastridae | 5 | 5.35 | 7.59 | 5 | 15 | +ve |
| Ostracoda | 39 | 29.93 | 31.04 | 30 | 65 | +ve |
| Palaemonidae | 82.5 | 28.31 | 33.33 | 50 | 95 | +ve |
| Parastacidae | 80 | 30.4 | 35.28 | 40 | 90 | +ve |
| Philopotamidae | 5 | 44.16 | 58.21 | 5 | 15 | -ve |
| Philorheithridae | 65 | 11.52 | 8.71 | 35 | 95 | -ve |
| Physidae | 15 | 28.34 | 23.74 | 12 | 40 | +ve |
| Planorbidae | 50 | 13.91 | 17.39 | 40 | 60 | +ve |
| Pleidae | 30 | 6.1 | 16.27 | 20 | 100 | +ve |
| Polycentropodidae | 5 | 7.32 | 11.96 | 0 | 13.1 | -ve |
| Protoneuridae | 15 | 5.76 | 9.96 | 5 | 30 | +ve |
| Psephenidae | 10 | 43.86 | 50.47 | 5.95 | 15 | -ve |
| Ptilodactylidae | 10 | 10.9 | 25.31 | 5 | 20 | -ve |
| Pyralidae | 5 | 8.57 | 7.41 | 0 | 10.025 | -ve |
| Richardsonianidae | 50 | 1.59 | 6.17 | 10 | 95 | +ve |
| Sciomyzidae | 80 | 1.85 | 10.62 | 44.75 | 100 | +ve |
| Sialidae | 20 | 5.91 | 11.92 | 10 | 40 | +ve |
| Simuliidae | 15 | 44.76 | 32.8 | 7.95 | 15 | -ve |
| Siphlonuridae | 0 | 1.29 | 5.16 | 0 | 40 | -ve |
| Spercheidae | 90 | 5.97 | 27.02 | 80 | 90 | +ve |
| Sphaeriidae | 10 | 7.64 | 7.47 | 10 | 63.1 | +ve |
| Sphaeromatidae | 20 | 1.54 | 5.53 | 0 | 50 | -ve |
| Staphylinidae | 90 | 10.63 | 17.06 | 40 | 100 | +ve |
| Stratiomyidae | 30 | 9.32 | 10.75 | 10 | 60 | +ve |
| Synlestidae | 5 | 14.84 | 12.16 | 5 | 10 | +ve |
| Tabanidae | 85 | 7.42 | 8.05 | 70 | 100 | +ve |
| Tasimiidae | 5 | 8.15 | 18.03 | 10 | 30 | -ve |
| Temnocephalidae | 100 | 8.98 | 15.16 | 30 | 100 | +ve |
| Thaumaleidae | 30 | 1.79 | 8.2 | 3 | 35 | -ve |
| Tipulidae | 10 | 36.63 | 32.29 | 10 | 15 | -ve |
| Veliidae | 25 | 50.46 | 32 | 10 | 30 | +ve |

Table S.6 Full Threshold Indicator Analysis (TITAN) results for Brazil taxa data. See Table S.5 for description of the result parameters.

|  | **zenv.cp** | **IndVal** | **zscore** | **5%** | **95%** | **filter** |
| --- | --- | --- | --- | --- | --- | --- |
| Baetidae | 42.64 | 53.58 | 5.54 | 41.17725 | 92.137 | -ve |
| Caenidae | 25.77 | 50.8 | 6.33 | 18.495 | 44.54025 | -ve |
| Calamoceratidae | 48.82 | 29.93 | 5.13 | 32.9745 | 92.46325 | -ve |
| Coenagrionidae | 28.205 | 53.61 | 5.92 | 26.235 | 43.94925 | -ve |
| Corixidae | 20.485 | 21.15 | 5.73 | 2.70125 | 45.37175 | -ve |
| Corydalidae | 53.54 | 30.12 | 6.08 | 46.388 | 96.0575 | -ve |
| Culicidae | 82.915 | 24.82 | 6.87 | 79.35 | 97.56 | +ve |
| Dolichopodidae | 6.2 | 46.31 | 8.58 | 5.41 | 44.22 | -ve |
| Euthyplociidae | 46.085 | 16.87 | 7.61 | 3.885 | 60.49225 | -ve |
| Glossosomatidae | 80.385 | 40.07 | 7.69 | 44.60575 | 87.1 | -ve |
| Hydropsychidae | 55.155 | 50.17 | 7.4 | 48.2 | 84.41325 | -ve |
| Hydroptilidae | 52.91 | 35.67 | 6.41 | 30.275 | 68.865 | -ve |
| Leptohyphidae | 48.59 | 52.2 | 5.49 | 34.495 | 87.06 | -ve |
| Leptophlebiidae | 42.64 | 51.22 | 6.74 | 33.51475 | 70.643 | -ve |
| Libellulidae | 24.12 | 52.82 | 4.38 | 16.665 | 53.4 | -ve |
| Megapodagrionidae | 53.14 | 23.5 | 7.75 | 10.5 | 58.63425 | -ve |
| Noteridae | 98.805 | 16.77 | 6.59 | 56.38 | 100 | +ve |
| Odontoceridae | 47.295 | 37.41 | 4.45 | 14.01575 | 84.04 | -ve |
| Perilestidae | 46.345 | 9.96 | 6.02 | 7.105 | 48.59 | -ve |
| Perlidae | 87.06 | 49.68 | 8.4 | 42.86 | 94.74 | -ve |
| Philopotamidae | 26.235 | 30.47 | 7.64 | 9.945 | 62.42825 | -ve |
| Planariidae | 42.545 | 22.44 | 4.48 | 24.716 | 72.42 | -ve |
| Planorbidae | 86.19 | 22.65 | 7.13 | 61.545 | 100 | +ve |
| Polycentropodidae | 12.255 | 59.6 | 6.48 | 9.76 | 44.20575 | -ve |
| Polythoridae | 29.79 | 13.12 | 8.78 | 17.64 | 32.02 | -ve |
| Psephenidae | 62.84 | 24.19 | 7.48 | 21.50875 | 71.0135 | -ve |
| Sialidae | 81.44 | 15.71 | 5.26 | 44.04825 | 100 | +ve |
| Simuliidae | 54.345 | 47.88 | 4.59 | 30.66325 | 92.865 | -ve |
| Tipulidae | 77.8 | 46.02 | 5.52 | 42.86 | 94.7535 | -ve |
| Veliidae | 77.035 | 33.04 | 4.37 | 40.9 | 99.685 | -ve |

Table S.7 Full Threshold Indicator Analysis (TITAN) results for the UK taxa data. See Table S.5 for description of the result parameters.

|  | **zenv.cp** | **IndVal** | **zscore** | **5%** | **95%** | **filter** |
| --- | --- | --- | --- | --- | --- | --- |
| Acarina | 95 | 24.87 | 13.75 | 15 | 98 | +ve |
| Acroloxidae | 44.5 | 6.28 | 12.27 | 19.8 | 100 | +ve |
| Arachnida | 15 | 39.02 | 13.68 | 10 | 30 | +ve |
| Asellidae | 28 | 47.44 | 28.26 | 20 | 35 | +ve |
| Astacidae | 85 | 4.3 | 4.88 | 5 | 100 | +ve |
| Athericidae | 30 | 11.32 | 8.67 | 0 | 30 | -ve |
| Baetidae | 20 | 63.94 | 29.15 | 15 | 30 | -ve |
| Beraeidae | 90 | 13.23 | 12.92 | 28 | 100 | +ve |
| Bithyniidae | 20 | 8.14 | 12.62 | 5 | 98 | +ve |
| Brachycentridae | 5 | 2.95 | 4.03 | 1 | 25 | +ve |
| Calopterygidae | 30 | 21.63 | 21.44 | 20 | 50 | +ve |
| Ceratopogonidae | 20 | 37.73 | 18.96 | 15 | 35 | +ve |
| Chironomidae | 60 | 62.59 | 9.45 | 47.975 | 70 | +ve |
| Chloroperlidae | 6 | 36.81 | 29.01 | 3 | 18 | -ve |
| Coenagrionidae | 83 | 16.55 | 21.39 | 35 | 90 | +ve |
| Collembola | 95 | 8.28 | 7.53 | 15 | 100 | +ve |
| Copepoda | 70 | 2.8 | 14.05 | 30 | 100 | +ve |
| Corixidae | 36.5 | 16.1 | 20 | 33.975 | 83.5 | +ve |
| Crangonyctidae | 65 | 20.09 | 15.09 | 50 | 99 | +ve |
| Culicidae | 75 | 5.2 | 16.52 | 39.425 | 80 | +ve |
| Dendrocoelidae | 25 | 11.63 | 14.88 | 6.95 | 50 | +ve |
| Diplostraca | 15 | 2.3 | 5.41 | 3 | 100 | +ve |
| Dixidae | 50 | 15.85 | 14.17 | 22 | 86.5 | +ve |
| Dryopidae | 3 | 1.17 | 2.61 | 2 | 20 | +ve |
| Dytiscidae | 20 | 32.12 | 14.72 | 20 | 39.05 | +ve |
| Elmidae | 70 | 66.93 | 12.54 | 50 | 89.5 | -ve |
| Empididae | 5 | 30.19 | 8.55 | 0 | 15.25 | -ve |
| Ephemerellidae | 5 | 32.12 | 11.09 | 1 | 5 | +ve |
| Ephemeridae | 15 | 29.53 | 22.7 | 10 | 27.525 | +ve |
| Ephydridae | 85 | 8.63 | 24.32 | 40 | 94.025 | +ve |
| Erpobdellidae | 30 | 31.42 | 13.45 | 15 | 35 | +ve |
| Gammaridae | 25 | 63.36 | 30.51 | 15 | 29 | +ve |
| Gerridae | 0 | 2.58 | 3.48 | 0 | 38.1 | +ve |
| Glossiphoniidae | 30 | 56.94 | 35.51 | 15 | 32.525 | +ve |
| Glossosomatidae | 3 | 39.91 | 14.68 | 1 | 5 | +ve |
| Goeridae | 2 | 44.43 | 16.66 | 2 | 7.05 | +ve |
| Gyrinidae | 28 | 25.33 | 7.47 | 19.85 | 70 | -ve |
| Haliplidae | 20 | 18.46 | 24.2 | 15 | 30 | +ve |
| Heptageniidae | 25 | 67.64 | 45.32 | 5 | 20.025 | -ve |
| Hydraenidae | 15 | 37.61 | 20.05 | 16 | 39 | -ve |
| Hydridae | 20 | 3.88 | 7.19 | 5 | 25 | +ve |
| Hydrobiidae | 5 | 21.91 | 15.17 | 5 | 30 | +ve |
| Hydrometridae | 63.5 | 5.13 | 34.42 | 50 | 85 | +ve |
| Hydrophilidae | 89.5 | 16.39 | 8 | 4 | 90 | +ve |
| Hydropsychidae | 20 | 59.36 | 21.43 | 5 | 30 | -ve |
| Insecta | 15 | 2.27 | 4.39 | 0 | 20 | -ve |
| Johnstonianidae | 5 | 0.81 | 2.74 | 4 | 78.525 | +ve |
| Lepidostomatidae | 28.5 | 33.62 | 13.46 | 25 | 32 | -ve |
| Leptoceridae | 25 | 31.21 | 10.73 | 3 | 25 | +ve |
| Leptophlebiidae | 10 | 25.64 | 7.39 | 5 | 100 | +ve |
| Leuctridae | 15 | 58.61 | 36.43 | 5 | 21.05 | -ve |
| Limnephilidae | 25 | 53.65 | 13.15 | 10 | 30 | +ve |
| Limoniidae | 25 | 22.55 | 12.62 | 10 | 58 | +ve |
| Lumbriculidae | 90 | 31.14 | 21.14 | 30 | 97.5 | +ve |
| Lymnaeidae | 25 | 30.12 | 9.07 | 12 | 30 | +ve |
| Microturbellaria | 20 | 2.31 | 5.95 | 4 | 80 | +ve |
| Molannidae | 65 | 2.24 | 6.52 | 10 | 75 | +ve |
| Nematoda | 7 | 8.88 | 9.1 | 2 | 20 | -ve |
| Nematomorpha | 13 | 1.98 | 4.5 | 2 | 20 | +ve |
| Nemouridae | 20 | 47.28 | 22.73 | 5 | 25 | -ve |
| Nepidae | 60 | 4.05 | 17.04 | 50 | 90 | +ve |
| Neuroptera | 20 | 0.86 | 3.73 | 5 | 75 | +ve |
| Niphargidae | 30 | 2.02 | 10.14 | 15 | 40 | +ve |
| Notonectidae | 80 | 12.8 | 35.18 | 50 | 90 | +ve |
| Odontoceridae | 30 | 15.06 | 6.26 | 4.85 | 45 | -ve |
| Oligochaeta | 4 | 53.13 | 8.36 | 1 | 5 | +ve |
| Ostracoda | 22 | 25.45 | 22.36 | 18.975 | 90 | +ve |
| Pediciidae | 80 | 40.35 | 4.07 | 0 | 95 | -ve |
| Perlidae | 2 | 25.44 | 29.07 | 2 | 5 | -ve |
| Perlodidae | 15 | 47.18 | 29.37 | 5 | 30 | -ve |
| Philopotamidae | 35 | 7.63 | 6.85 | 5 | 40 | -ve |
| Phryganeidae | 20 | 1.44 | 10.08 | 17.475 | 70 | +ve |
| Physidae | 68.5 | 33.32 | 23.48 | 60 | 80 | +ve |
| Piscicolidae | 20 | 17.74 | 25.14 | 15 | 25 | +ve |
| Planorbidae | 2 | 41.37 | 9.31 | 1 | 5 | +ve |
| Polycentropodidae | 20 | 32.22 | 11.08 | 10 | 25 | -ve |
| Psychodidae | 50 | 16.77 | 6.07 | 25 | 90 | +ve |
| Psychomyiidae | 90 | 43.77 | 13.67 | 27 | 100 | +ve |
| Ptychopteridae | 60 | 21.27 | 28.08 | 30 | 85 | +ve |
| Pyralidae | 85 | 6.06 | 15.26 | 60 | 90 | +ve |
| Rhyacophilidae | 15 | 59.02 | 32.34 | 15 | 30 | -ve |
| Sciomyzidae | 70 | 3.58 | 12.76 | 25 | 90 | +ve |
| Sericostomatidae | 5 | 46.2 | 19.32 | 1 | 7.05 | +ve |
| Sialidae | 25 | 21.58 | 21.48 | 20 | 35 | +ve |
| Simuliidae | 30 | 54.89 | 12.04 | 20 | 75 | -ve |
| Sphaeriidae | 15 | 68.11 | 43.67 | 15 | 30 | +ve |
| Stratiomyidae | 30 | 10.58 | 12.2 | 23 | 35 | +ve |
| Succineidae | 50 | 8.79 | 17.9 | 25 | 60 | +ve |
| Tabanidae | 35 | 6.4 | 12.93 | 30 | 98.5 | +ve |
| Taeniopterygidae | 25 | 14.74 | 8.55 | 5 | 27 | -ve |
| Tateidae | 20 | 31.71 | 20.98 | 15 | 32.55 | +ve |
| Tubificidae | 45 | 15.71 | 19.74 | 35 | 90 | +ve |
| Valvatidae | 20 | 16.28 | 20.47 | 20 | 83 | +ve |
| Veliidae | 40 | 10.43 | 18.21 | 34.475 | 98 | +ve |

Table S.8 Full Threshold Indicator Analysis (TITAN) results for New Zealand taxa data. See Table S.5 for description of the result parameters.

|  | **zenv.cp** | **IndVal** | **zscore** | **5%** | **95%** | **filter** |
| --- | --- | --- | --- | --- | --- | --- |
| Austroperlidae | 5.375 | 17.49 | 9.35 | 0.86875 | 20 | -ve |
| Calocidae | 0 | 37.06 | 4.06 | 0 | 87.5 | -ve |
| Cladocera | 100 | 41.56 | 8.32 | 32.5 | 100 | +ve |
| Coenagrionidae | 86.25 | 34.07 | 10.58 | 37.475 | 87.5 | +ve |
| Collembola | 87.5 | 19.52 | 4.47 | 5 | 100 | +ve |
| Coloburiscidae | 60 | 29.72 | 7.57 | 5 | 80.0625 | -ve |
| Conoesucidae | 60 | 58.86 | 8.16 | 30 | 88.75 | -ve |
| Copepoda | 87.5 | 55.85 | 13.85 | 60 | 98.575 | +ve |
| Corixidae | 40 | 28.7 | 5.92 | 18.125 | 60 | +ve |
| Corydalidae | 64.375 | 39.55 | 8.82 | 10 | 87.5 | -ve |
| Culicidae | 81.25 | 10.58 | 6.3 | 20 | 87.5 | +ve |
| Dugesiidae | 96.75 | 22.86 | 13.95 | 87.5 | 100 | +ve |
| Dytiscidae | 40 | 19.13 | 5.94 | 15.5 | 87.5 | +ve |
| Elmidae | 71.25 | 71.25 | 13.56 | 20 | 72.5 | -ve |
| Eustheniidae | 5 | 24.52 | 10.92 | 1.375 | 15.375 | -ve |
| Gripopterygidae | 14.375 | 38.65 | 10.62 | 1.5 | 30 | -ve |
| Helicopsychidae | 60 | 26.16 | 4.51 | 18.05625 | 86.3125 | -ve |
| Hydraenidae | 5.125 | 28.21 | 8.15 | 0 | 60 | -ve |
| Hydridae | 20 | 7.23 | 2.8 | 6.49375 | 100 | +ve |
| Hydrobiosidae | 76.25 | 67.6 | 11.4 | 30 | 86.25 | -ve |
| Hydropsychidae | 85 | 52.03 | 9.15 | 30 | 86.25 | -ve |
| Hydrozoa | 96.75 | 17.05 | 14.94 | 87.5 | 100 | +ve |
| Isotomidae | 92 | 20.17 | 14.52 | 87.5 | 100 | +ve |
| Leptophlebiidae | 60 | 75.36 | 17.28 | 20 | 68.15625 | -ve |
| Limoniidae | 32.5 | 48.24 | 9.28 | 1.86875 | 60 | -ve |
| Lymnaeidae | 100 | 20.21 | 7.01 | 38.86875 | 100 | +ve |
| Nematoda | 92 | 82.6 | 18.82 | 87.5 | 100 | +ve |
| Nemertea | 25.75 | 19.6 | 4.24 | 5 | 60 | +ve |
| Nesameletidae | 10 | 28.92 | 8.15 | 0 | 60 | -ve |
| Notonectidae | 40 | 11.28 | 7.13 | 34.5 | 87.5 | +ve |
| Odonata | 60 | 10.12 | 4.46 | 34.2375 | 87.5 | +ve |
| Oeconesidae | 0 | 27.17 | 8.77 | 0 | 60 | -ve |
| Oligochaeta | 60 | 60.29 | 7.14 | 51.2375 | 96.5 | +ve |
| Ostracoda | 61.875 | 62.14 | 11.01 | 33.2125 | 98.2625 | +ve |
| Paracalliopidae | 87.5 | 34.71 | 7.24 | 38.21875 | 100 | +ve |
| Philopotamidae | 0 | 44.25 | 4.81 | 0 | 77.5625 | -ve |
| Physidae | 87.5 | 53.9 | 9.62 | 29.5 | 87.5 | +ve |
| Planorbidae | 53.75 | 32.33 | 7.18 | 50 | 100 | +ve |
| Platyhelminthes | 29.5 | 28.23 | 4.29 | 5 | 87.5 | +ve |
| Sarcophagidae | 60 | 14.11 | 6.51 | 20 | 60 | +ve |
| Sciomyzidae | 100 | 27.36 | 8.73 | 61.78125 | 100 | +ve |
| Sphaeriidae | 92 | 65.67 | 12.67 | 62.46875 | 100 | +ve |
| Tateidae | 20 | 66.74 | 11.18 | 5 | 39.5 | +ve |
| Veliidae | 10 | 36.42 | 7.07 | 6.5 | 87.5 | +ve |

Table S.9 Full Threshold Indicator Analysis (TITAN) results for Australian trait data. See Table S.5 for description of the result parameters. Trait abbreviations in Supplementary 1 Table S.3.

|  | **zenv.cp** | **IndVal** | **zscore** | **5%** | **95%** | **filter** |
| --- | --- | --- | --- | --- | --- | --- |
| feed_filter | 12 | 67.7 | 40.93 | 10 | 30 | -ve |
| feed_gatherer | 7 | 51.98 | 9.16 | 5 | 10 | -ve |
| feed_herbivore | 25 | 59.72 | 33.97 | 20 | 55 | -ve |
| feed_predator | 15 | 64.2 | 44.06 | 5 | 20 | +ve |
| feed_shredder | 5 | 60.9 | 19.09 | 0 | 5 | +ve |
| resp_gil | 30 | 55.57 | 40.62 | 15 | 80 | -ve |
| resp_pls_spi | 10 | 59.99 | 46.87 | 10 | 20 | +ve |
| resp_teg | 10 | 62.47 | 29.32 | 10 | 20 | -ve |
| locom_burrow | 15.5 | 68.9 | 52.19 | 14.95 | 40 | -ve |
| locom_crawl | 80 | 52.82 | 19.01 | 35 | 90 | -ve |
| locom_sessil | 12 | 70.42 | 65.9 | 10 | 20 | -ve |
| locom_swim | 10 | 64.07 | 40.73 | 10 | 20 | +ve |
| size_large | 15 | 64.31 | 34.24 | 10 | 20 | -ve |
| size_medium | 1 | 55.49 | 18.71 | 0 | 6.05 | +ve |
| size_small | 60 | 52.79 | 14.61 | 55 | 98.525 | +ve |
| ovip_aqu | 95 | 52.16 | 20.04 | 0 | 98 | -ve |
| ovip_ovo | 70 | 7.78 | 6.41 | 36.975 | 80 | -ve |
| ovip_ter | 15 | 65.22 | 37.02 | 8 | 20 | +ve |
| volt_bi_multi | 40 | 53.18 | 29.74 | 10 | 60 | +ve |
| volt_semi | 10 | 52.41 | 5.92 | 10 | 20 | -ve |
| volt_uni | 60 | 58.59 | 36.72 | 40 | 90 | -ve |

Table S.10 Full Threshold Indicator Analysis (TITAN) results for Brazil trait data. See Table S.5 for description of the result parameters. Trait abbreviations in Supplementary 1 Table S.3.

|  | **zenv.cp** | **IndVal** | **zscore** | **5%** | **95%** | **filter** |
| --- | --- | --- | --- | --- | --- | --- |
| feed_gatherer | 62.26 | 56.6 | 6.06 | 34.21 | 94.4955 | -ve |
| feed_herbivore | 79.38 | 57.46 | 6.09 | 35.08075 | 86.67225 | -ve |
| feed_parasite | 92.12 | 59.28 | 3.63 | 31.15175 | 100 | -ve |
| feed_predator | 79.38 | 67.31 | 10.26 | 41.95 | 86.715 | +ve |
| resp_gil | 100 | 57.23 | 3.71 | 22.51925 | 100 | -ve |
| resp_pls_spi | 79.38 | 69.03 | 6.29 | 41.4195 | 87.022 | +ve |
| locom_burrow | 84.04 | 68.69 | 9.1 | 37.66 | 86.71725 | +ve |
| locom_crawl | 85.045 | 53.43 | 7.39 | 80.72 | 98.14075 | -ve |
| size_large | 79.38 | 65.54 | 7.98 | 41.9495 | 84.387 | +ve |
| size_medium | 40.9 | 55.7 | 3.26 | 26.416 | 100 | +ve |
| size_small | 79.38 | 54.38 | 8.79 | 75.57 | 92.865 | -ve |
| ovip_ter | 42.86 | 50.83 | 3.79 | 41.1 | 94.22825 | +ve |
| volt_bi_multi | 79.885 | 55.58 | 5.94 | 38.55 | 94.82 | -ve |
| volt_semi | 79.38 | 65.02 | 7.31 | 30.52 | 82.47525 | +ve |

Table S.11 Full Threshold Indicator Analysis (TITAN) results for the UK trait data. See Table S.5 for description of the result parameters. Trait abbreviations in Supplementary 1 Table S.3.

|  | **zenv.cp** | **IndVal** | **zscore** | **5%** | **95%** | **filter** |
| --- | --- | --- | --- | --- | --- | --- |
| feed_shredder | 3 | 54.75 | 7.85 | 0 | 10 | +ve |
| feed_gatherer | 35 | 55.67 | 16.75 | 20 | 70 | +ve |
| feed_herbivore | 70 | 60.05 | 23.85 | 35 | 90 | -ve |
| resp_gil | 80 | 52.6 | 7.44 | 2 | 90 | -ve |
| resp_pls_spi | 3.5 | 61.67 | 21.16 | 2 | 7 | +ve |
| locom_sessil | 2 | 59.04 | 12.69 | 2 | 10 | +ve |
| locom_swim | 15 | 56.22 | 16.89 | 5 | 25 | -ve |
| locom_crawl | 43.5 | 51.01 | 7.85 | 15 | 94 | +ve |
| size_medium | 5 | 56.61 | 23.95 | 3 | 20 | -ve |
| size_small | 1 | 53.56 | 21.31 | 1 | 5 | +ve |
| size_large | 30 | 64.63 | 27.01 | 25 | 45 | +ve |
| ovip_aqu | 74 | 53.3 | 27.38 | 60 | 90 | -ve |
| ovip_ovo | 30 | 63.41 | 20.26 | 20 | 45 | +ve |
| ovip_ter | 10 | 60.84 | 15.48 | 7 | 30 | +ve |
| volt_semi | 15 | 59.52 | 14.33 | 9 | 35 | +ve |
| volt_uni | 65 | 53.74 | 11.91 | 35 | 74.5 | -ve |
| volt_bi_multi | 65 | 54.82 | 7.45 | 35 | 75 | +ve |

Table S.12 Full Threshold Indicator Analysis (TITAN) results for New Zealand trait data. See Table S.5 for description of the result parameters. Trait abbreviations in Supplementary 1 Table S.3.

|  | **zenv.cp** | **IndVal** | **zscore** | **5%** | **95%** | **filter** |
| --- | --- | --- | --- | --- | --- | --- |
| feed_shredder | 92 | 63.83 | 5.43 | 87.5 | 100 | -ve |
| feed_gatherer | 12.75 | 35.13 | 8.34 | 0 | 15 | -ve |
| feed_herbivore | 50 | 53.45 | 7.77 | 8.75 | 60 | -ve |
| resp_teg | 92 | 58.77 | 8.77 | 20 | 96.5 | +ve |
| resp_gil | 60 | 61.47 | 10.88 | 29.975 | 88.8125 | -ve |
| locom_swim | 37.625 | 59.54 | 8.41 | 10 | 67.75 | +ve |
| locom_crawl | 60 | 53.63 | 10.77 | 50 | 87.5 | -ve |
| locom_burrow | 92 | 57.84 | 3.59 | 0 | 100 | +ve |
| size_large | 88.75 | 48.18 | 5.12 | 1.5 | 96.5 | -ve |
| ovip_ter | 92 | 64.27 | 6.29 | 10 | 100 | -ve |
| volt_uni | 92 | 68.87 | 15.95 | 87.5 | 97.0625 | -ve |
| volt_bi_multi | 60 | 65.58 | 11.12 | 20 | 87.5 | +ve |
